# Optogenetic approaches addressing extracellular modulation of neural excitability

**DOI:** 10.1038/srep23947

**Published:** 2016-04-05

**Authors:** Emily A. Ferenczi, Johannes Vierock, Kyoko Atsuta-Tsunoda, Satoshi P. Tsunoda, Charu Ramakrishnan, Christopher Gorini, Kimberly Thompson, Soo Yeun Lee, Andre Berndt, Chelsey Perry, Sonja Minniberger, Arend Vogt, Joanna Mattis, Rohit Prakash, Scott Delp, Karl Deisseroth, Peter Hegemann

**Affiliations:** 1 Bioengineering, Stanford University, 318 Campus Drive, Stanford, CA 94305, USA; 2 Neurosciences, Stanford University, 318 Campus Drive, Stanford, CA 94305, USA; 3 Institute of Biology, Experimental Biophysics, Invalidenstraße 42, D-10115 Berlin, Germany; 4 HHMI, Stanford University, 318 Campus Drive, Stanford, CA 94305, USA; 5 Department of Psychiatry & Behavioral Science, Stanford University, 401 Quarry Road, Stanford, CA 94305, USA

## Abstract

The extracellular ionic environment in neural tissue has the capacity to influence, and be influenced by, natural bouts of neural activity. We employed optogenetic approaches to control and investigate these interactions within and between cells, and across spatial scales. We began by developing a temporally precise means to study microdomain-scale interactions between extracellular protons and acid-sensing ion channels (ASICs). By coupling single-component proton-transporting optogenetic tools to ASICs to create two-component optogenetic constructs (TCOs), we found that acidification of the local extracellular membrane surface by a light-activated proton pump recruited a slow inward ASIC current, which required molecular proximity of the two components on the membrane. To elicit more global effects of activity modulation on ‘bystander’ neurons not under direct control, we used densely-expressed depolarizing (ChR2) or hyperpolarizing (eArch3.0, eNpHR3.0) tools to create a slow non-synaptic membrane current in bystander neurons, which matched the current direction seen in the directly modulated neurons. Extracellular protons played contributory role but were insufficient to explain the entire bystander effect, suggesting the recruitment of other mechanisms. Together, these findings present a new approach to the engineering of multicomponent optogenetic tools to manipulate ionic microdomains, and probe the complex neuronal-extracellular space interactions that regulate neural excitability.

The extracellular ionic environment in neural tissue plays a critical role in regulating the resting membrane potential and signaling events such as action potential generation^1^,^2^. Although the brain appears designed to homeostatically maintain a constant extracellular milieu, neurons may experience fluctuations in the concentration of extracellular ions (e.g. K^+^ or H^+^) during epochs of altered neural activity^3^,^4^,^5^,^6^,^7^. Microbe-derived optogenetic tools that regulate transmembrane ionic flux have been developed and expressed in genetically-specified cell types; these tools modulate the activity of expressing cells with millisecond temporal precision, causing depolarization in the case of cation-conducting channelrhodopsins (such as ChR2)^8^,^9^, or hyperpolarization in the case of chloride pumps (halorhodopsins such as NpHR)^10^ and proton pumps (bacteriorhodopsins and archaerhodopsins such as Arch)^11^,^12^. However, the full extent of influence of these tools on the extracellular ionic environment remains to be explored^13^,^14^. Here we investigate the role of extracellular ions in modulating neural excitability, both in the gating of ion channels at the extracellular surface of the membrane, and in mediating the interaction between activity-modulated neurons and their neighbors.

Extracellular protons evoke multiple currents in primary afferent neurons, which are carried by several acid-sensitive ion channels^15^,^16^,^17^. In the central nervous system (CNS) these channels are involved in nociception^18^, synaptic transmission^17^ and taste reception^19^. Many neuronal membrane proteins are modulated by extracellular protons such as acid-sensing ion channels (ASICs)^17^, acid-sensitive TASK potassium channels^20^, and NMDA receptors^21^. ASICs contribute to the excitatory postsynaptic current by modulating the density of dendritic spines^22^ and synaptic plasticity^23^,^24^. They have been implicated in fear-related learning and memory^25^, seizure termination^26^ and a variety of neuropsychiatric syndromes^27^. Among these channels involved in acid-sensing activity, acid sensitive ion channels (ASICs) and transient receptor potential vallinoid sensitive ion channels (TRPVs) have been most thoroughly studied. ASICs belong to the voltage insensitive, amiloride-sensitive epithelial Na^+^-channels/degenerin family of cation channels^28^. The proton-sensitive members of this family that are expressed in mammals are encoded by four different genes which are alternatively spliced to produce six subunit isoforms: ASIC1a, ASIC1b, ASIC2a, ASIC2b, ASIC3 and ASIC4^17^,^29^,^30^,^31^. Most ASICs respond to moderate decreases in extracellular pH^30^, unlike TRPV, which is activated only by severe acidosis (pH < 6)^32^,^33^. ASICs were therefore our primary choice for sensing the pH gradient generated by a light-activated proton pump upon illumination.

Properties of ASIC channels such as pH sensitivity, kinetics and ion selectivity have previously been characterized in *Xenopus laevis* oocytes. The pH required for activation and particularly the kinetics for activation, inactivation, and desensitization at low pH are very different among individual homo- and hetero-multimers^30^,^34^,^35^. A key leap forward in the understanding of ASIC function was achieved through resolving the crystal structure of chicken ASIC1a at 1.9 Å resolution, which has greater than 90% homology to its human and rat counterparts^31^. Each subunit of the functional trimer is characterized by two membrane spanning helical sequences, a large cysteine-rich extracellular loop and short intracellular N- and C-termini. The structure of the extracellular domain resembles a clenched hand with five subdomains that is linked to the transmembrane part via a flexible wrist. Inter-subunit interaction is extensive but no ion-conducting path is visible. Since the protein was crystallized at low pH the structure resembles the desensitized state. The proton binding residues are distant from the channel region and protons are thought to induce a major conformational change in the protein^31^.

To investigate the functional characteristics of the interaction between extracellular protons and neural excitability, we developed strategies for coupling optogenetic (light-mediated) extracellular proton extrusion to activation of proton-gated ion channels, choosing ASICs for their large conductance, and selectivity for a single ion species (see refs 36 and 37 for related approaches). In our approach, we aimed to determine whether extrusion of protons can influence local neural activity in a spatially-constrained fashion on the same membrane, and whether activity-induced transmembrane ion fluxes can exert a longer range influence on the excitability of neighboring expressing cells. Although both natural and optogenetically-driven activity patterns can continue to influence the intrinsic excitability of cells after the offset of the activity change itself^13^,^14^, such an excitability influence on non-directly-modulated nearby “bystander neurons” has yet to be explored using optogenetics as a tool.

## Results

### The development of two-component optogenetics (TCO) to explore H^+^-ASIC interactions on the extracellular membrane surface

**Figure 1:**
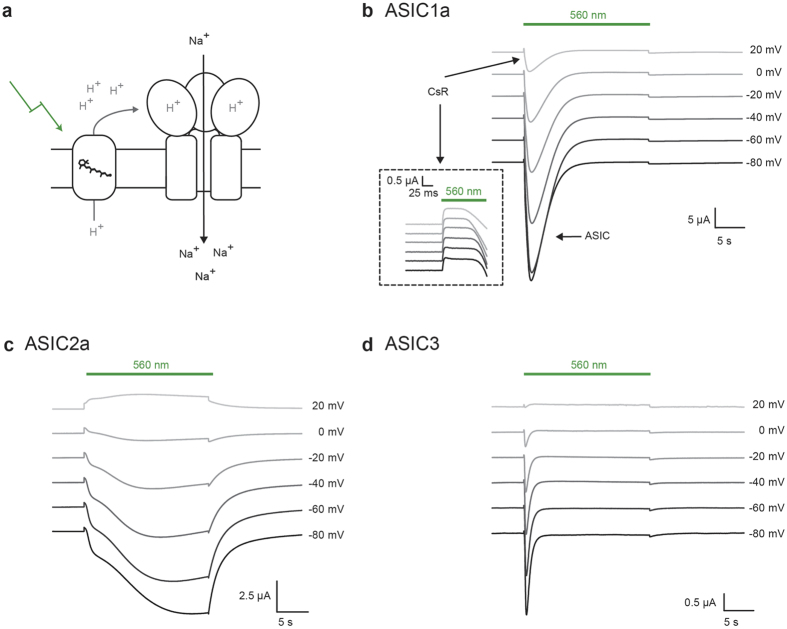
Light-activation of three acid-sensitive ion channels. (**a**) Principle of the two-component optogenetic (TCO) approach. Upon illumination the light-activated proton pump may moderately acidify the local extracellular medium and activate acid-sensitive ion channels, ASICs, via their proton-sensing domain. This results in a remote but large sodium influx that can be used for sustained cell depolarization at moderate light intensities. In *Xenopus* oocytes, a light-driven proton pump of Coccomyxa subellipsoidea (CsR) was used. (**b–d**) Macroscopic currents of CsR_T46N_ coexpressed with rat ASIC1a (**b**), rat ASIC2a (**c**) or rat ASIC3 (**d**) in oocytes at a molar RNA ratio of 1:1 (for ASIC3 of 2:1). Cells were illuminated with 560 nm light at different holding voltages at 0.1 mM MOPS and pH 7.5 under constant perfusion. The small outward directed pump currents (CsR) triggers large inward sodium currents (ASIC). Inset is a zoom-in to the initial pump activity directly after starting to illuminate CsR_T46N_-ASIC1a with green light. Note that ASIC1a and ASIC3 show strong inactivation in sustained light, whereas ASIC2a shows moderate to no inactivation at all.

We began by exploring the effects of extracellular protons on neural membrane excitability by developing an optogenetic approach for generating local proton microdomains using two-component optogenetics. Extracellular proton-gated ion channels, known as acid sensing ion channels (ASICs), are widely expressed in the peripheral and central nervous system. We hypothesized that activation of ASICs requires the formation of local pockets or “microdomains” of decreased pH as there is evidence that this may occur physiologically at synapses^38^, where ASICs are concentrated and have been implicated in synaptic transmission^24^,^39^,^40^. To test this, we developed a modular system in which a light-sensitive protein such as a proton pump is co-expressed with a secondary-coupled ion channel, such as an acid-sensing ion channel (ASIC), to evoke a light-triggered secondary current carried by a specific ionic species, (Fig. 1a). In designing the two-component approach in *Xenopus leavis* oocytes, we chose a light-driven proton pump of the arctic green alga *Coccomyxa subellipsoidea* C-169 (CsR)^41^, which has improved expression in oocytes compared to the well-characterized bacteriorhodopsin or archaerhodopsin, currently used for hyperpolarization of neurons^11^,^12^. We used the CsR mutant T46N, which exhibits less voltage dependence than the wild type, allowing larger photocurrents at negative voltages (Supplementary Fig. 1).

In *Xenopus leavis* oocytes we co-expressed CsR with each of three different rat acid-sensing ion channels ASIC1a, ASIC2a or ASIC3. These channels are characterized by steep pH-dependence of the proton-activated currents below physiological pH^17^,^30^,^42^. Immediately after light onset we observed a small outward current mediated by proton pumping of CsR followed by a large inward current carried by the co-expressed acid-sensing ion channel (Fig. 1b–d). For both ASIC1a and ASIC3, the secondary activated inward current peaked within 1–2 s after light onset and then rapidly decayed to the initial pump current, due to the high proton sensitivity and fast desensitization of ASIC1a and ASIC3 as described previously^30^ (Fig. 1b,d). In contrast ASIC2a mediated a long-lasting light activated inward current (Fig. 1c). The rise of the ASIC2a current was multiphasic at all voltages, a property not observed in previous studies in which the channel was directly activated by sudden acidification of the bulk solution. This is possibly due to the indirect activation of the channel by the proton pump. In accordance with a low pH_50_ of 5 and the reported slow and incomplete desensitization of ASIC2a^30^, the light-induced currents decayed only very slowly during illumination. Following light offset the current decayed to zero within ~20 seconds and could be reactivated by illumination at any time (Supplementary Fig. 2).

**Figure 2:**
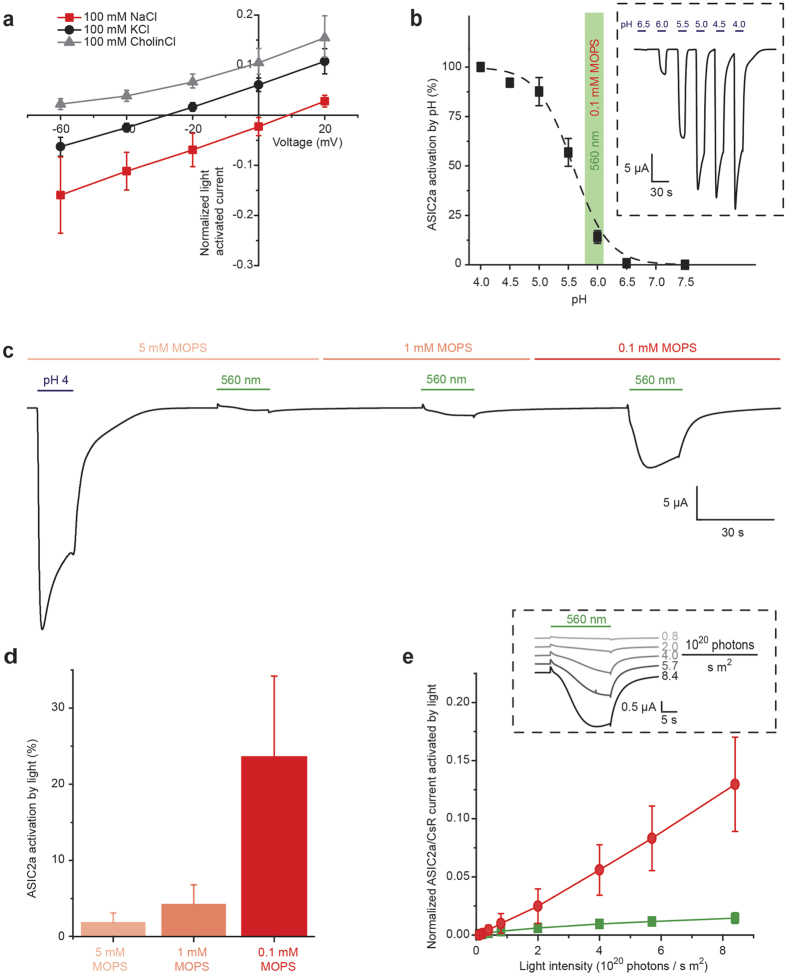
Characterization of CsR-ASIC2a by two-electrode voltage clamp (TEVC) recordings in oocytes. (**a**) Current-voltage dependency of normalized photocurrents in 100 mM NaCl, 100 mM KCl or 100 mM CholineCl extracellular medium (all media contained additionally 1mM NaCl/KCl, 1 mM MgCl_2_, 0.1 mM CaCl_2_ and 0.1 mM MOPS, pH 7.5, normalized to ASIC2a current activated by pH 4, mean +/− SD, n = 5). (**b**) ASIC2a currents measured during pH titration in darkness and comparison with photocurrents measured at pH 7.5 (mean +/− SD, n = 6). The green shaded region highlights the percent activation of ASIC2a by illumination with green light at 0.1 mM MOPS at −40 mV (data shown in Fig. 5d). Inset: representative pH activated current trace of ASIC2a at −40 mV. (**c**) Macroscopic currents of CsR_T46N_-ASIC2a activated by pH 4 or green light at different buffer concentrations (5 mM MOPS, 1 mM MOPS and 0.1 mM MOPS, −40 mV, constant perfusion). (**d**) Percent activation of ASIC2a by the light driven proton pump CsR_T46N_ in different buffer concentrations (5 mM MOPS, 1 mM MOPS and 0.1 mM MOPS, −40 mV, n = 9, 100% activation taken as the peak ASIC current produced by pH 4, mean +/− SD, n = 9). (**e**) Normalized ASIC2a (red data points) and CsR_T46N_ (green data points) photocurrents measured at different light intensities (0.1 mM MOPS, −40 mV, normalized to ASIC2a current activated by pH 4, mean +/− SD, n = 5). Inset: representative current traces at −40 mV.

The observed light-activated inward currents were inversely proportional to the membrane voltage due to the increased driving force for Na^+^ at negative voltages and voltage independent gating and permeability of ASIC2a^30^ (Figs 1c and 2a). Changing the concentration of the major extracellular cation from Na^+^ to K^+^ or choline shifted the reversal potential and greatly decreased the magnitude of the inward current component, confirming the Na^+^-selectivity of the ASIC2a response as a major feature of the TCO construct (Fig. 2a). To determine the fraction of ASIC2a channels that were activated by light, we titrated the ASIC2a currents by rapid buffer exchange. We found that the maximal current (when holding the membrane potential at −40 mV) was only reached at pH 4 (Fig. 2b). Normalization of light-activated currents to this value revealed that approximately 25% of ASICs are activated by CsR-mediated acidification (Fig. 2b–d) and allowed an approximation of the acidification sensed by ASICs at the cell surface (Fig. 2b green bar). We also noticed that at pH values of 5.5 or below, ASIC2a inactivates more severely than at pH 6. Thus for neuronal applications, the degree of inactivation may serve as a useful indicator for the external pH value that may have been reached.

ASIC activation strongly depended on the number of active proton pumps as probed by application of light at different intensities (Fig. 2e). Furthermore, efficient activation depended on the external buffering capacity for protons, namely buffer strength and extracellular volume. Increasing the buffer strength from 0.1 mM to 5 mM strongly decreased the ASIC2a mediated inward current (Fig. 2c) and correspondingly the fraction of light activated ASIC2a channels from ~25% at 0.1 mM MOPS to ~2% at 5 mM MOPS (Fig. 2d). In contrast, the extracellular bulk volume exhibited lesser importance; consecutive exchange of the bulk medium during illumination by continuous perfusion only slightly decreased the light activated ASIC2a current compared to conditions with a constant bulk phase (Supplementary Fig. 2). In a weakly buffered solution (0.1 mM MOPS), recovery of the ASIC2a-mediated inward current was much slower than after direct activation of ASIC2a by acidic solution (pH 4) (Supplementary Fig. 3). This supports the concept that in a weakly buffered extracellular medium, CsR causes a persistent acidification of volumes on the cell surface, thus maintaining ASIC channels in an inactivated state and slowing down peak current recovery.

### Two-component optogenetics in neural tissue

**Figure 3:**
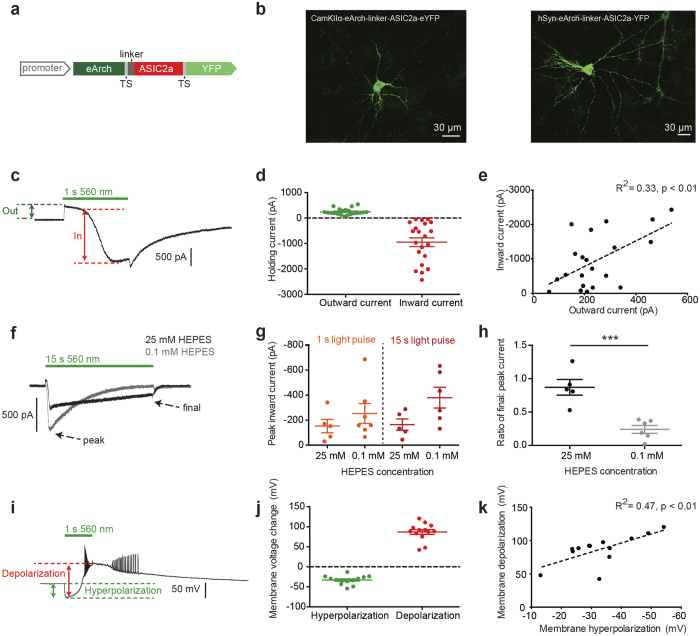
eArch3.0-ASIC2a (Channel and Pump, Champ) expression in cultured hippocampal neurons. (**a**) Two-component Champ2.0 construct containing eArch (enhanced by trafficking sequence, TS) and ASIC2a, separated by a linker sequence and labeled with YFP. (**b**) Champ2.0 expression under CamKIIα or human synapsin (hSyn) promoters. (**c**) Representative voltage clamp trace for a Champ-expressing cell in response to a 1 s pulse of 560 nm light (green horizontal line). (**d**) Magnitude of outward (mean +/− SEM = 246 +/− 27 pA) and inward (−950 +/− 172 pA) components of the Champ current to a 1 s pulse of 560 nm light (n = 21). (**e**) Relationship between inward and outward components of the current (n = 21). Linear regression analysis yields R^2^ = 0.33, p = 0.006 for difference of slope from zero (F(1, 19) = 9.447). (**f**) Example Champ responses to a 15 s light pulse in standard (25 mM HEPES, black trace) and weakly buffered (0.1 mM HEPES, grey trace) extracellular solution. (**g**) Peak inward currents in 25 and 0.1 mM HEPES for 1 s and 15 s light pulses. For 15 s light pulses, mean inward current is −164 pA +/−45 at 25 mM (n = 5) versus −379.9 +/−83 pA at 0.1 mM (n = 6), unpaired t-test with Welch’s correction: t = 2.278, df = 7.558, p = 0.0541). (**h**) Decay of inward current (final to peak current ratio for 15 s light pulse) in 25 mM (n = 5) versus 0.1 mM HEPES (n = 6), unpaired t-test with Welch’s correction: t = 4.779, df = 5.997, p = 0.0031. (**i**) Example membrane potential response to 1 s pulse of 560 nm light (green horizontal line) for a Champ expressing cell recorded in current clamp. (**j**) Magnitude of hyperpolarizing (eArch3.0-mediated, −33 +/− 3 mV) and depolarizing (ASIC2a-mediated, 87 +/− 6 mV) components of the light response (n = 13). (**k**) Relationship between Champ-mediated membrane hyperpolarization and depolarization (for 1 s light pulses). Linear regression analysis yields R^2^ = 0.47, p = 0.0093 for difference of slope from zero (F(1, 11) = 9.885).

ASIC2a was chosen to assess the interaction between extracellular pH and ASICs in cultured hippocampal neurons. The channels were co-expressed with the light-driven, trafficking-enhanced proton pump eArch3.0, one of the highest-expressing pumps in neurons^11^,^12^. We developed a single eArch3.0-ASIC2a construct, enhanced for better membrane localization with trafficking signal (TS) and endoplasmic reticulum (ER) export motifs^43^. This construct was named Champ (channel and pump), fusing the proton pump and ASIC2a at the DNA level. The two proteins were separated either by a p2a ribosomal skip sequence (Champ1.0) or linked by a short amino acid linker sequence (Champ2.0 and 3.0). We performed whole-cell patch clamp recordings from cultured hippocampal pyramidal neurons, expressing Champ constructs under the human synapsin (hSyn) or Ca^2+^/calmodulin-dependent kinase IIα (CaMKIIα) promoters (Champ2.0 shown here, Fig. 3a,b) and observed a characteristic biphasic membrane current in response to 560 nm light in voltage-clamp recordings (Fig. 3c). The mean current magnitudes were 246 pA for the outward proton pump-mediated component and −950 pA for the inward ASIC-mediated component (Fig. 3d). The biphasic current was observed in 48% of YFP-positive neurons (Supplementary Fig. 4a,b) and there was no physiological evidence of adverse effects on cell health in neurons expressing the dual component construct (Supplementary Fig. 4c–f).

The inward current magnitude was (as a first approximation) linearly related to the magnitude of the outward proton pump current (Fig. 3e). Peak current magnitudes did not vary significantly with the duration of light pulses (1–15 s) (Supplementary 5a,b), suggesting that maximal membrane currents were achievable within 1 s, however off-kinetics, as fitted by a two-term exponential, increased with increasing light pulse duration (Supplementary Fig. 5b). For longer light pulses (15 s), we observed a clear decay in the inward current magnitude over the course of the light pulse to approximately 80% of the initial value (Fig. 3f,h), which may be explained by increases in extracellular acidity and ASIC desensitization under sustained light conditions. We wondered whether the properties of the ASIC-mediated current would be influenced by extracellular buffering capacity. In a weakly buffered solution (0.1 mM HEPES) 7/11 (~60%) neurons exhibited the characteristic biphasic membrane response, compared to 5/10 (50%) neurons in standard buffer strength (25 mM), under otherwise matched conditions. The depolarizing currents tended to be larger in the weakly buffered solution, nearly doubling during longer light pulses (15 s) (p = 0.054) (Fig. 3g) however this was accompanied by a decrease in the stability of the response with a pronounced decay of the peak inward current over the duration of the light pulse (Fig. 3f,h), likely due to the faster spatial spread of protons away from the membrane due to faster desensitization at more acidic extracellular pH.

Current clamp recordings of membrane potential responses of TCO-expressing neurons revealed that 560 nm light evoked an initial hyperpolarization (−33 mV) of the membrane potential, followed by a subsequent depolarization (87 mV) (Fig. 3i,j) which was sufficient to generate action potentials (~9 spikes) and persisted beyond the termination of the light pulse. The extent of ASIC-mediated depolarization was proportional to the initial pump-mediated hyperpolarization (Fig. 3k), echoing the linear relationship between the inward and outward current components seen in voltage-clamp recordings. The light pulse duration did not significantly alter the magnitude of membrane potential change or number of spikes elicited (Supplementary Fig. 5c,d).

We questioned whether prolonged pumping of protons out of the cell by eArch3.0 could modulate the probability of Ca^2+^-dependent activation of EF-hand Ca^2+^-binding proteins through intracellular alkalization^44^. Immunostaining for activation of downstream signaling pathways (phosphorylated CREB, phosphorylated MAPK, and cFos) in light-exposed neurons did not reveal differences in the levels of activation of these proteins in Champ-expressing neurons compared to YFP-expressing controls, but which were significantly lower than positive control neurons exposed to a high K^+^-containing Tyrode’s solution, confirming that the Champ construct did not perturb intracellular Ca^2+^ signaling either through direct Ca^2+^ entry or through intracellular alkalinization (Supplementary Fig. 6).

### The role of pH microdomains in ASIC activation revealed by two-component optogenetics

**Figure 4:**
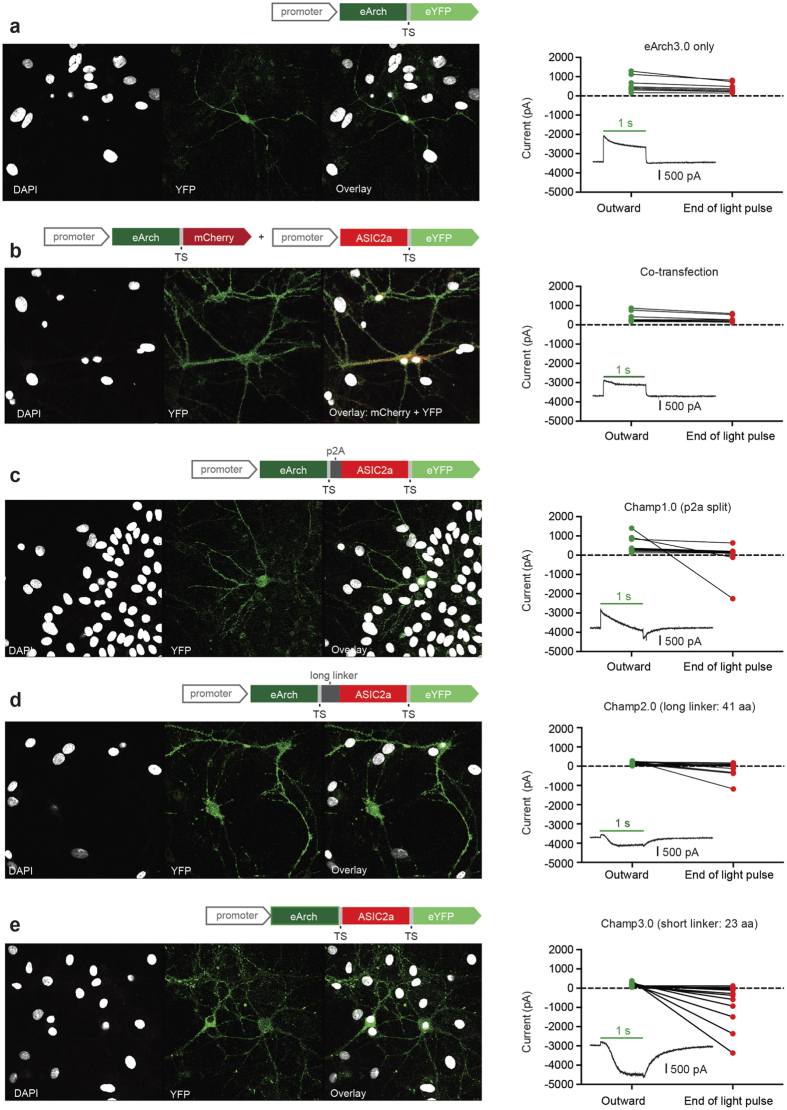
Head-to-head comparison of four different Champ constructs in which the proton pump and ASIC are separated by increasing primary-sequence distance. For each construct: a cartoon illustrates the structure of the two-component construct, confocal images demonstrate fluorescence expression in culture and graphs show the relative magnitude of the peak outward current and the current at the end of the light pulse. A more negative current at the end of the light pulse indicates a larger ASIC component. Insets: representative traces of the current responses to a 1 s pulse of 560 nm light for each two-component construct (timing of light pulse indicated by green horizontal line). All electrophysiological recordings were performed in low HEPES (0.1 mM) Tyrode’s solution. (**a**) eArch3.0-YFP only control (n = 9). (**b**) Co-transfection of eArch3.0 and ASIC2a: eArch3.0 is labeled with mCherry and ASIC2a is labeled with YFP to allow identification of both components in a single cell (n = 9). (**c**) Champ1.0: eArch3.0 and ASIC2a are separated during protein translation by the ribosomal skip sequence, p2A (n = 14). (**d**) Champ2.0: eArch3.0 and ASIC2a are fused by a 41 amino acid linker sequence (n = 17). (**e**) Champ3.0: eArch3.0 and ASIC2a are fused by a short linker sequence (23 amino acid membrane trafficking signal, TS) (n = 16).

Having demonstrated the importance of extracellular buffering, we hypothesized that the spatial extent of proton spread might be important for the observed response. To test this, we systematically altered the molecular separation of the two components. In the first construct (Champ1.0), a ribosomal p2a skip sequence was placed between the two components thus completely cleaving the two proteins during translation^45^,^46^. In a subsequent construct described previously (Champ2.0), a 41 amino acid linker sequence (trafficking sequence plus additional inert amino acids) tethered the two proteins. We compared this to a third construct (Champ3.0) with a shorter linker sequence (23 amino acid trafficking sequence only), which fused the two proteins more closely. As a control, we co-transfected neurons simultaneously with two separate constructs (CaMKIIα-Arch3.0-mCherry and CamKIIα-ASIC2a-YFP). We tested the 4 constructs and an additional CaMKIIα-eArch3.0-YFP control in a head-to-head comparison under matched experimental conditions. All four approaches resulted in good YFP expression, indicating successful expression of the ASIC construct. In the co-expression experiment we also saw good co-localization of mCherry fluorescence confirming expression of both constructs within single cells (Fig. 4). We noticed that a shorter molecular separation between the constructs resulted in a higher probability of observing the inward current component of the TCO response. When the proteins were simply co-expressed, no inward ASIC-mediated current was observed, and outward current magnitudes were similar to the eArch3.0 control currents (Fig. 4a,b). When the proteins were split by the p2a sequence, we observed occasional large ASIC-mediated inward currents (Fig. 4c). As the molecular separation between the components decreased by fusion with a linker sequence (Champ2.0), the size and reliability of ASIC-induced inward currents increased (Fig. 4d), with cells expressing the shortest linker sequence (TS) construct (Champ3.0) exhibiting the most consistent and largest ASIC-mediated inward currents (Fig. 4e). The observed dependence of TCO function on the apparent physical proximity of the proton pump and ASIC channel highlights the importance of microenvironment ion sensing in the interaction between the two components on the membrane.

**Figure 5:**
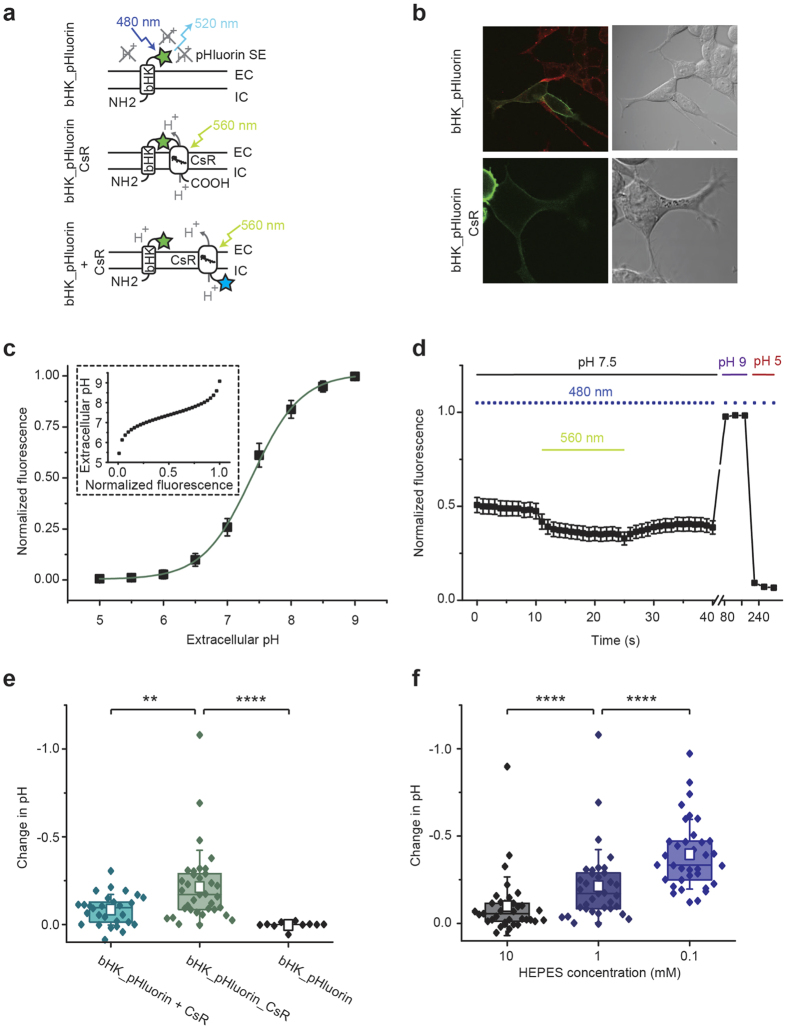
pH changes at the extracellular membrane surface in HEK293 cells. (**a**) Protein architectures of bHK_pHluorin (ß-subunit of the HK-ATPase fused to superecliptic pHluorin), bHK_pHluorin_CsR (bHK_pHluorin fused to the N-terminus of CsR T46N) and bHK_pHluorin + CsR (bHK_pHluorin coexpressed with CsR_T46N_eCFP via a P2A cleavage site). (**b**) Confocal images of bHK_pHluorin and bHK_pHluorin_CsR (pHluorin in green, Rhodamine18 membrane marker in red (only for bHK_pHluorin)). (**c**) Normalized fluorescence of bHK_pHluorin as a function of extracellular pH (mean +/− SD, n = 9). Inset: Extracellular pH dependence on normalized fluorescence. (**d**) Experimental protocol for the measurement of extracellular pH changes: bHK_pHluorin_CsR was illuminated for 15 s by 560 nm light with simultaneous fluorescence measurements of pHluorin (480 nm excitation pulses) (mean +/− SEM, n = 25), calibrated by fluorescence measurements at pH 9 and pH 5. (**e**) Extracellular pH changes after 560 nm light in 1 mM HEPES in absence of a proton pump (bHK_pHluorin, n = 10), in direct vicinity to a proton pump (bHK_pHluorin_CsR, n = 35) and on the overall membrane surface in presence of CsR_T46N, (bHK_pHluorin + CsR, n = 27) (box represents 25% to 75% percentile, empty square represents mean + − SD, two sample t-test with Welch’s correction with t = −3.1l, df = 47.7, p = 0.0032 for the comparison of bHK_pHluorin + CsR to bHK_pHluorin_CsR and t = 5.97, df = 36.17, p < 0.0001 for the comparison of bHK_pHluorin_CsR to bHK_pHluorin). **(f**) pH changes in direct vicinity to CsR T46N measured with bHK_pHluorin_CsR in different extracellular proton buffer concentrations (n = 35, two-sample paired t-test with t = −6.24, df = 34, p < 0.0001 for the comparison of 10 mM HEPES to 1 mM HEPES and t = −7.03, df = 34, p < 0.0001 for the comparison of 1 mM HEPES to 0.1 mM HEPES). Photocurrents of the proton pump constructs were comparable but variable and overall small reflecting the small pH changes observed with the fluorescent dye (45 +− 30 pA for bHK_pHluorin_CsR and 55 +− 45 pA for the CsR_eCFP_P2A_bHK_pHluorin split construct).

To further test this concept, we performed imaging experiments with a membrane-tethered pH sensor to measure pH changes at different distances from the proton pump (Fig. 5a–d). In HEK cells, pH changes detected by a membrane-tethered pHluorin were on average more prominent if the sensor was directly fused to the proton pump, rather than co-translationally cleaved by a p2a skip sequence (Fig. 5e). The extracellular acidification generated by the pHluorin_CsR fusion construct was greater with decreasing concentration of HEPES buffer (Fig. 5f), suggesting that proton microdomains are formed through a combination of fast proton diffusion along the membrane and acidification of extracellular diffusion-limited volumes.

### Activity-dependent (non-synaptic) modulation of local neural activity: the bystander effect

**Figure 6:**
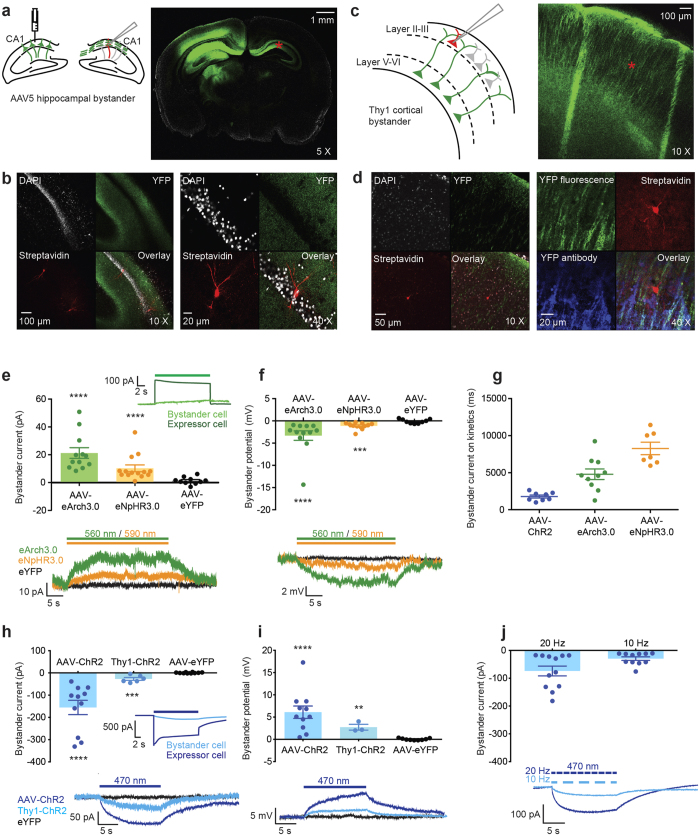
The bystander effect. (**a**) Unilateral injection of AAV5-CamKII-(opsin)-eYFP into CA1 of hippocampus yielded non-expressing bystander neurons in the contralateral hippocampus (location represented by red star). (**b**) Biocytin-filled hippocampal bystander neurons in CA1. (**c**) Bystander neurons in superficial cortex of Thy1-ChR2 (line 18) transgenic mice (location represented by red star). (**d**) Bystander neuron surrounded by but not overlapping with YFP expression. (**e**) Hyperpolarizing bystander currents (30 s 560 nm light) for eArch3.0 (21 +/− 4 pA, n = 12, p < 0.0001), 590 nm for eNpHR3.0 (10 +/− 2 pA, n = 14, p < 0.0001) and YFP controls (1.3 +/− 0.8 pA, n = 10, 560 nm). Inset: Comparison with eArch3.0 photocurrent. (**f**) Hyperpolarizing bystander potentials for eArch3.0 (−3.3 +/− 1.1 mV, n = 12, p < 0.0001, eNpHR3.0 (−1.1 +/− 0.2 mV, n = 12, p < 0.001, and YFP controls (−0.1 +/− 0.2 mV, n = 9). (**g**) Onset kinetics (τ_on_) for depolarizing (ChR2: 1800 +/− 200 ms, n = 8) and hyperpolarizing (eArch3.0: 4800 +/− 710 ms, n = 10, eNpHR3.0: 8300 +/− 850 ms, n = 7) bystanders. (**h**) Depolarizing bystander currents (15 s 470 nm light) for ChR2 hippocampal bystanders (mean +/− SEM = −155 +/− 32 pA, n = 11, p < 0.0001), Thy1-ChR2 cortical bystanders (−27 +/− 6 pA, n = 6, p < 0.001) and YFP controls (0.7 +/− 0.7 pA, n = 10). Inset: Comparison with ChR2 photocurrent. (**i**) Depolarizing bystander potentials for ChR2 hippocampal bystanders (6.1 +/− 1.4 mV, n = 11, p < 0.0001), Thy1-ChR2 bystanders (2.7 +/− 0.6 mV, n = 3, p < 0.01) and YFP controls (0.02 +/− 0.07 mV, n = 9). Bystander currents for 470 nm pulse trains at 20 Hz (−73 +/− 18 pA, n = 12) and 10 Hz (−29 +/− 6 pA, n = 11). Statistical comparison are between opsin and YFP control groups using the Mann Whitney unpaired t-test. Data are from a total of 22 animals.

Having demonstrated the ability of light-activated proton pumps to modulate neural excitability through changes in ion concentration at the extracellular membrane surface, we next tested the ability of these optogenetic tools (when densely expressed and powerfully driven) to modulate activity in neighboring non-expressing neurons via precisely timed transmembrane ionic fluxes (“bystander neurons” were defined as neurons exposed to, but not directly expressing, optogenetically-mediated changes in local neural activity). To record from bystander neurons, we employed two different optogenetic targeting strategies. First, we separately expressed different optogenetic constructs (eArch3.0, eNpHR3.0, ChR2_H134R_ or a YFP control), driven by the calmodulin kinase IIα promoter (AAV5-CamKIIα-(opsin)-eYFP), unilaterally in the CA1 region of hippocampus. Capitalizing on the contralaterally-projecting axons of these neurons, we then recorded from non-expressing neurons (bystander neurons) densely surrounded by opsin-expressing axons in the contralateral CA1 (in the presence of blockers of ionotropic synaptic transmission to inhibit fast synaptic influences of optogenetic manipulations; Fig. 6a,b). Second, we employed the transgenic mouse strain Thy1-ChR2 (line 18), wherein neocortical ChR2 is expressed largely in layer V cortical neurons^47^ to record from non-expressing bystander neurons located in superficial cortical layers densely surrounded by ChR2-expressing membrane processes but not expressing ChR2 themselves (Fig. 6c,d).

We first examined whether eArch3.0 would cause depolarization of bystander neurons, as observed in neurons expressing the Champ constructs. Unexpectedly, during a 30 s light pulse, bystanders of proton-pumping neurons (AAV5-eArch3.0 expressing) instead exhibited a very small (mean = 21 pA) and slow (τ_on _~ 5 s) hyperpolarizing current (Fig. 6e–g) rather than the secondary depolarization observed in Champ expressing cells, wherein eArch3.0 activated covalently-linked ASIC2a channels. We questioned whether a similar phenomenon would be seen with optogenetic neural silencers pumping other ionic species. In bystanders of chloride-pumping cells (AAV5-eNpHR3.0-expressing) we again observed a small hyperpolarizing current (mean = 10 pA) with very slow kinetics (τ_on _~ 8 s) (Fig. 6e–g). These tiny outward currents corresponded to a small mean membrane hyperpolarization of −3.3 mV for eArch3.0 and −1.1 mV for eNpHR3.0 whereas no change in membrane current or voltage was observed for AAV5-YFP control bystanders under matched experimental conditions (Fig. 6f).

Having demonstrated that hyperpolarizing tools generated a slow hyperpolarizing current in bystander neurons, we hypothesized that depolarizing optogenetic tools could produce the opposite effect. Indeed, in response to a 15 s blue light pulse, hippocampal ChR2 bystander neurons exhibited a small depolarizing membrane current under these conditions (mean = −155 pA) (Fig. 6h). This bystander current had onset-kinetics several orders of magnitude slower (τ_on _~ 2 s) than a direct ChR2 photocurrent (Fig. 6h inset), even when ChR2 was weakly expressed (Supplementary Fig. 8a–c). We noted however, that these slow kinetics (on the order of seconds) (Fig. 6g), were reminiscent of the kinetics of ASIC-mediated currents that we had measured previously (Supplementary Fig. 2d). Cortical Thy1-ChR2 bystander neurons displayed a smaller inward current (mean =−27 pA) (Fig. 6h) consistent with the smaller direct ChR2 photocurrent magnitude in Thy1-ChR2-expressing neurons (Supplementary Fig. 8d). These inward currents corresponded to a mean membrane depolarization of 6.1 mV for hippocampal ChR2 bystanders and 2.7 mV for cortical Thy1-ChR2 bystanders (Fig. 6i). AAV5-YFP bystanders did not exhibit a change in membrane current or voltage. Consistent with this effect being recruitable by native activity patterns, bystander effects were observed in response to modest 20 or even 10 Hz light pulse trains with similar slow inward currents (mean = −73 pA for 20 Hz and −29 pA for 10 Hz) (Fig. 6j).

During light application, ChR2 bystander neurons experienced a 24% mean decrease in membrane resistance, whereas eArch3.0-bystander neurons experienced a 9% increase in membrane resistance and eNpHR3.0 a 2% increase. YFP controls showed a 3% decrease in membrane resistance (Supplementary Fig. 9a). Current-voltage relationships were unusual, exhibiting a significantly positive slope for depolarizing bystanders and significantly negative slope for hyperpolarizing bystanders, converging at a reversal potential between −10 to −40 mV (Supplementary Fig. 9b), and raising the possibility of activation as well as inactivation of several intrinsic ion channels of different ion selectivity. At rest, bystander neurons did not show evidence of adverse cell health relative to neurons surrounded by inert YFP-control expressing membranes (Supplementary Fig. 9c–e).

### Functional and mechanistic exploration of the bystander effect

**Figure 7:**
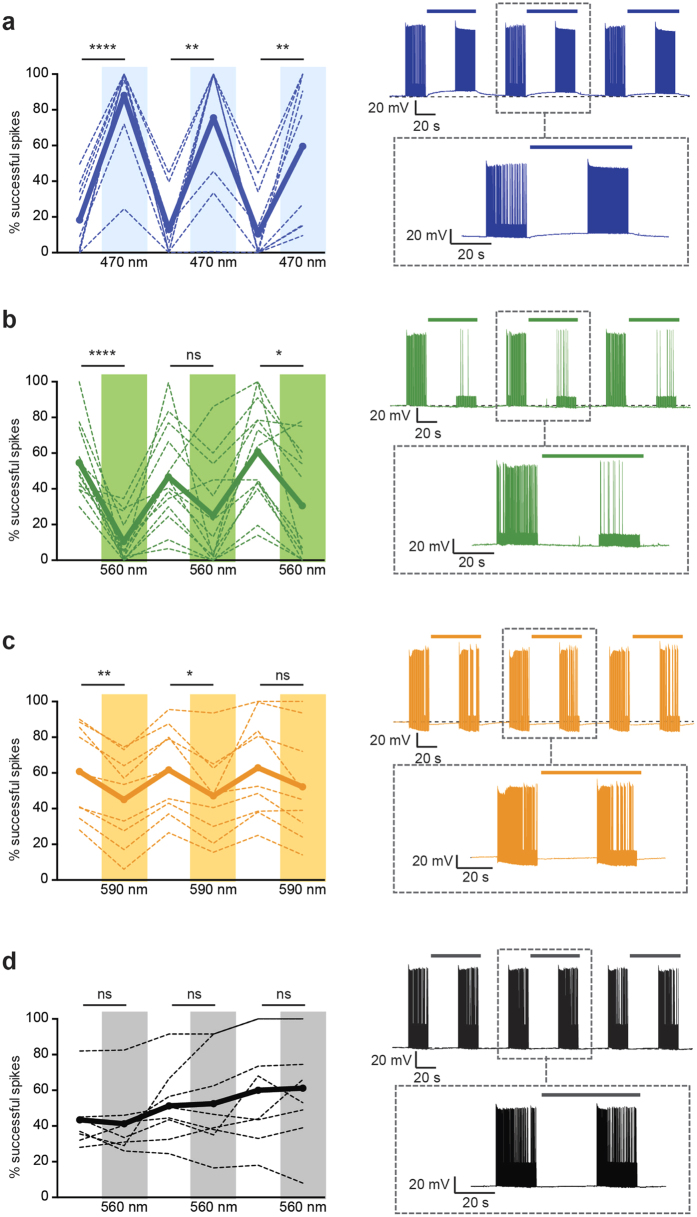
Functional impact of bystander currents in neurons at spike threshold. To facilitate effect detection, spikes were evoked in the bystander neuron by intracellular injection of electrical current pulses at 10 Hz with current magnitudes titrated to spike threshold, to achieve ~50% spike success rate at baseline. In dense opsin-expressing regions, light stimulation was then applied and the change in evoked spiking of bystander neurons was recorded. Plots show percentage of successfully evoked spikes during repeated light-off and light-on epochs for (**a**), AAV-ChR2 (RM one-way ANOVA: F = 28.78, n = 10 cells, p < 0.0001). (**b**) AAV-eArch3.0 (F = 12.16, n = 13 cells, p < 0.0001). (**c**) AAV-eNpHR3.0 (F = 8.361, n = 9, p = 0.030). (**d**) AAV-YFP control bystander neurons (F = 2.813, n = 8 cells, p = 0.1197). In summary plots (left), thick lines indicate group mean and thin lines indicate individual cell data. Example traces are shown (right) with dashed box containing zoom-in of the center light-off/light-on epoch. For bystander neurons in which baseline spike success was not near threshold, these significant effects of light on evoked spiking were not observed (not shown). Statistical comparisons were performed using a repeated measures one-way ANOVA (for 6 alternating treatments, 3 light off, 3 light on) with Tukey’s multiple comparison test. Asterisks represent significant differences after multiple comparison correction. Significance thresholds were set at p < 0.05 (*), p < 0.01 (**), p < 0.001 (***) and p < 0.0001 (****). Data are from a total of 23 animals.

We questioned whether the bystander effect could influence evoked action potential firing in non-directly-controlled neurons (Fig. 7). Although bystander currents were relatively small, even under high opsin-expression conditions, for proof-of-principle demonstration we set the current injection into recorded neurons right at action potential-initiation threshold to maximize the possibility of detecting any effect on spiking. During epochs of illumination (470 nm, 560 nm or 590 nm), the proportion of action potentials evoked in spike threshold-poised bystander neurons could indeed be modulated in a bidirectional manner for AAV5-ChR2 bystanders (Fig. 7a) and AAV5-eArch3.0 bystanders (Fig. 7b). AAV5-eNpHR3.0 bystanders also experienced a more modest but detectable reduction in spiking success during illumination (Fig. 7c) whereas no modulation by light epochs was observed in AAV5-YFP controls (Fig. 7d), and inhibition or driving of spiking was not observed when the bystander cell membrane potential was not poised near spike threshold. Having observed that the bystander effect tracked the direction of change in local neural activity we investigated whether such an effect could also be observed during non-optically driven neural activity changes. Although electrical stimulation is not constrained to a genetically specified cell type or projection and the effects are typically hard to isolate and study^48^, we endeavored to create “electrical bystanders” by stimulating axonal inputs (Schaffer collaterals) to the CA1 region of hippocampus (Supplementary Fig. 10a). We confirmed the ability of the stimulation paradigm to induce synaptic release (Supplementary Fig. 10b), and applied ionotropic synaptic transmission blockers to isolate the impact of axonal activity on the extracellular milieu. To mimic our optogenetic manipulations, we used 0.5–5 mA extracellular current pulses at a frequency of 10 Hz for a period of 15 s (Supplementary Fig. 10c,d). Following just a single stimulation epoch, the mean current immediately post-stimulation-artifact compared to baseline was significantly more negative than for non-modulated neurons (−11 pA) (Supplementary Fig. 10e) and displayed a slow recovery reminiscent of the ChR2 optogenetic bystander currents. We performed the experiments using both tungsten and platinum-iridium electrodes, finding similar effects with both electrode-types (Supplementary Fig. 10c,d). Hence bystander-like currents can be detected even with moderate non-optical stimulation.

**Figure 8:**
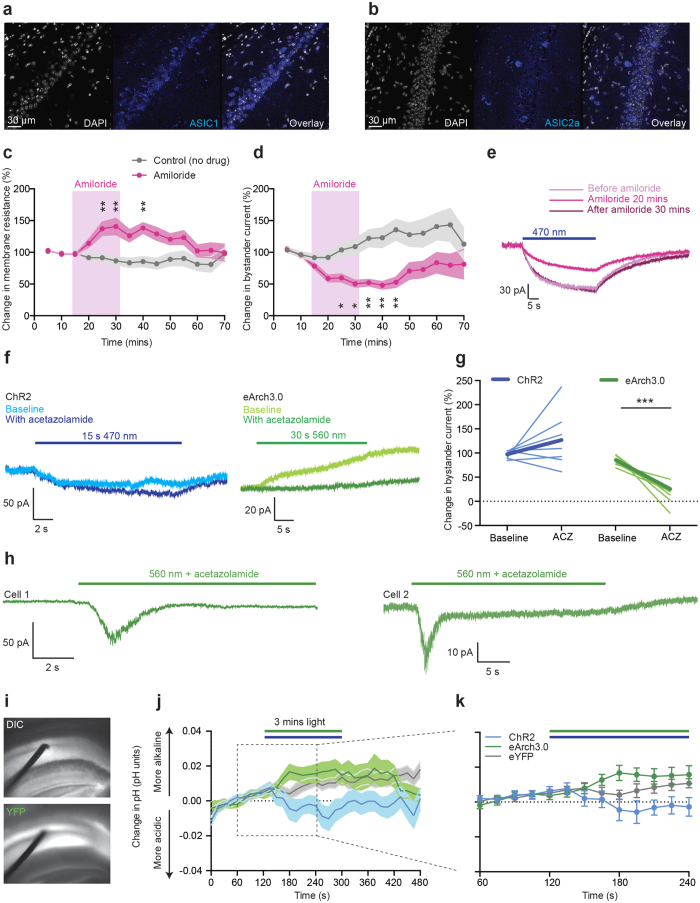
The contribution of acid-sensing ion channels to the bystander effect. (**a**,**b**) ASIC1 (**a**) and ASIC2a (**b**) expression in CA1. (**c**) Change in membrane resistance during amiloride (ASIC antagonist) application (lilac-shaded region) (n = 6–18 cells, 9 mice, two-way ANOVA for interaction between time and drug treatment (F (12,314) = 2.617, p = 0.0018, asterisks indicate significant time points after Dunnet’s multiple comparison test). Grey data points: control experiment in which no amiloride was applied (n = 4–16, 6 mice) (no significant change from baseline). (**d**) Change in bystander current during amiloride application (F (12,314) = 2.473, p = 0.0032, asterisks indicate significant time points). No significant change from baseline for untreated control cells. (**e**) Examples bystander current at baseline baseline (dark red), after 20 mins amiloride (pink), and after 30 mins of washout (lilac). (**f**) Example ChR2 and eArch3.0 bystander currents in 500 μM acetazolamide (pale traces indicate baseline recordings, dark traces indicate 15 mins acetazolamide exposure). (**g**) Change in bystander current magnitude after acetazolamide application (thick line represents group mean) (ChR2, n = 7 cells, 3 mice, paired t-test: t = 1.313, df = 6, p = 0.2372; eArch3.0, n = 7 cells, 3 mice, paired t-test: t = 6.177, df = 6, p = 0.0008). (**h**) Occasionally, acetazolamide caused eArch3.0 bystander neurons to exhibit inward ASIC-like currents during green light. (**i**) Extracellular pH measurements in CA1 (contralateral to site of opsin injection) in acute slices using a solid state metal wire oxide pH sensor (100 μm diameter, 5X magnification). (**j**) pH change in response to three minutes of light stimulation (470 nm for ChR2 and YFP-controls, 560 nm for eArch3.0). For ChR2, n = 24 recording sites, 13 slices, 3 animals. For eArch3.0, n = 21 recording sites, 11 slices, 3 animals. For YFP controls, n = 22 recording sites, 12 slices, 2 animals. (**k**) Zoom-in of last minute of baseline pH recording and first two minutes of light stimulation.

Since previous studies have shown that prolonged neural activity can be associated with decreases in extracellular pH^49^,^50^,^51^ and we had found the ChR2-induced bystander current to be depolarizing during ChR2 activation, with kinetics on the order of seconds, reminiscent of ASIC currents, we questioned whether ASIC activation could play a contributory role. We first confirmed the expression of both ASIC1 and ASIC2a in CA1 region of hippocampus using immunohistochemistry (Fig. 8a,b). We next tested the contribution of ASICs to the ChR2 bystander current using pharmacological blockade by the ASIC inhibitor, amiloride^17^. We evoked a bystander current (15 s light pulse) in hippocampal ChR2 bystander neurons every 5 minutes for up to 70 minutes. Following two baseline measurements, we applied 500 μM amiloride^52^ for 20 minutes, then returned to ACSF alone for a washout period. During amiloride application, we observed an increase in membrane resistance (in the absence of any illumination, suggesting that a proportion of ASICs may be open at rest) (Fig. 8c, Supplementary Fig. 11b) with a concurrent reduction in the magnitude of the light-evoked bystander current to ~50% of the baseline value, which slowly recovered after amiloride was washed out (Fig. 8d,e) suggesting that the bystander effect initiated by ChR2 is at least partially caused by ASIC modulation. Since ASIC activation is caused by extracellular pH reduction unlikely to be mediated by ChR2 itself (since ChR2 is only a very weak proton conductance regulator^53^ under physiological conditions), a potential acidification is most likely mediated by other factors^49^,^50^,^51^.

Due to the contributory role of ASIC in ChR2 bystander currents, we questioned whether ASIC inactivation could play a role in eArch3.0 hyperpolarizing bystander currents. However, such hyperpolarization would be unexpected, since proton pumps transport protons to the extracellular space and we would expect an extracellular acidification and subsequent depolarizing ASIC activation as seen in our Champ experiments. To address this dichotomy, we further analyzed extracellular pH changes and the impact of the extracellular proton buffer strength on bystander currents (Fig. 8). Interestingly, when the extracellular buffering capacity was reduced by the carbonic anhydrase inhibitor, acetazolamide (Fig. 8f–h) the eArch3.0 bystander current decreased or even inverted from outward (hyperpolarizing) to inward (depolarizing) (Fig. 8h) without impairing bystander cell health (Supplementary Fig. 11). These inward bystander currents were transient and reminiscent of ASIC1a currents, consistent with the concept that bicarbonate-buffering prevents ASIC activation of eArch3.0 bystander neurons by spatially limiting the spread of extracellular protons extruded by eArch3.0, such that the resultant bystander current is dominated by other secondary mechanisms. In support of this interpretation, pH-sensitive microelectrode recordings did not reveal significant acidification of the extracellular space during hippocampal eArch3.0 activation under bicarbonate buffering conditions (Fig. 8i–k). The secondary mechanisms noted above remain to be elucidated, though it is anticipated that the connection between actuator eArch3.0 expressors and bystander neurons may be of a predominantly ephaptic nature under bicarbonate-buffer conditions. Only when buffering capacity is reduced, the extruded extracellular protons may spread further, and in some cases in sufficient quantities to activate ASICs (likely ASIC1a) on neighboring neurons. In the case of the excitatory activity-bystander relationship (as with electrical and channelrhodopsin stimulation), there appears to exist an additional contribution from ASIC activation under normal buffering conditions, perhaps mediated by localized extracellular acidification at the synapse (where ASIC2 channels tend to congregate^38^) secondary to the activity of bystander channels, transporters or vesicular release of protons from the synaptic terminal, all as would also occur in the course of native synaptic activity.

## Discussion

Here we have developed an approach to the engineering of multicomponent tools to manipulate ionic microdomains, and to probe the complex neuronal-extracellular space interactions that regulate neural excitability. We identify distinct extracellular phenomena at different spatial scales: the paracrine effects of high local proton concentrations at the extracellular membrane surface, and longer-range ephaptic-like effects of neural activity on surrounding neural membranes (Supplementary Fig. 12).

On a very local scale in close vicinity to the cell surface the development of a two-component optogenetic (TCO) approach allowed us to causally investigate the ability of extracellular proton microdomains, created by the pumping action of light-sensitive proton pumps, to activate secondary coupled ion channels. We show that acid-sensitive ion channels (ASICs) respond to light-mediated extracellular acidification in host cells such as *Xenopus laevis* oocytes and cultured hippocampal neurons, when they are co-expressed (oocytes) or fused (neurons) with a light-driven ion pump, such as CsR or eArch3.0. Consistent with our initial expectations, the extracellular buffer strength and relative expression levels of the proton pump and ASIC appeared to be key determinants of current amplitudes and kinetics, with a ~1:1 ratio of CsR/eArch3.0:ASIC2a working well in both oocytes and hippocampal neurons.

Efficient activation of ASIC2a by the proton pump in oocytes under constant perfusion could occur via two potential mechanisms. The high degree of invagination of the stage IV oocytes limits the diffusion access to the oocyte membrane^54^,^55^ and could account for small diffusion-limited “micro-environments” which could be rapidly acidified by CsR and activate ASIC. In addition, long-range proton transfer along the membrane surface that is faster than or at least competing with proton exchange with the bulk water phase^56^,^57^ could also account for ASIC activation. Both mechanisms are in agreement with the high dependence of the current on the extracellular buffer strength observed in *Xenopus laevis* oocytes. First, case elevating the buffer strength increases the number of protons required for sufficient acidification of the diffusion limited extracellular environment. Second, case H^+^-buffering decreases the range of proton transfer along the membrane by facilitating proton exchange with the bulk phase. Both interpretations were supported by light-driven activation of ASIC2a in neurons under physiological conditions. Covalently linking the proton-pumping component, eArch3.0, to the acid-sensing component, ASIC2a, first co-localized the pump and the channel to the same extracellular environment and second, diminishing the intermolecular distance between the two components, highly improved functional ASIC2a activation.

These observations support the interpretation that proton-extrusion-mediated acidification is highly local and inhomogeneous along the cell surface; also consistent with this interpretation is that eArch3.0 did not induce depolarizing ASIC-like currents in bystander neurons under normal buffering conditions, but only when buffering capacity was reduced with acetazolamide (Fig. 8h), suggesting that extracellular buffering systems could be key determinants in the generation of proton microdomains generated by any proton extrusion mechanism including light-activated proton pumps. Such an interpretation does not exclude proton transfer to the bulk phase, but such protons are not converted into a significant bulk pH-change. This dichotomy of proton surface migration and bulk phase transfer was confirmed by imaging the extracellular pH changes evoked by the proton pump CsR at different distances from a membrane-tethered pH sensor (pHluorin) in HEK cells. Co-localization of the proton pump to the pH sensor, as well as a weakly buffered extracellular solution, enhanced the pH changes detected at the membrane, supporting the hypothesis that proton microdomains require both fast proton transfer along the membrane in addition to acidification of local extracellular volumes, the spatial extent of which are regulated by the extracellular buffering capacity.

It is important to note that there was significant variability in the response of the two-component construct, Champ, to light stimulation in neurons (Supplementary Fig. 4). Potential reasons for this could include variable expression level of the construct in individual neurons, variability in the location of the construct on the membrane, (which could influence the ability of a microdomain to form, for example near a branch point versus a smooth membrane region). It is also possible that exogenously expressed ASIC2a subunits interact with native ASIC subunits, bringing eArch3.0 into contact with a larger number of ASIC channels at physiologically relevant locations. In addition, it has been shown that ASIC2 subunits may help target ASIC channel expression to synapses through their association with synaptic proteins (e.g. PSD-95^38^).

In addition to this local paracrine modulation of neural activity by protons, we used projection-targeted, bidirectional optogenetic tools to demonstrate the existence of a longer-range, slow bystander neuron membrane current. This current followed the direction of local neural activity and was of sufficient magnitude under conditions of dense surrounding expression to influence action potential frequency in bystander neurons at spike threshold (recruiting a mechanism likely also recruited by natural (non-optically elicited) neural activity). ASIC modulation was found to play a role in these currents, but surprisingly was more relevant for the ChR2 stimulation environment (where ASIC channel activation contributed approximately 50% of the bystander current) than for eArch3.0. However, the observation that both depolarizing (ChR2) and hyperpolarizing (eArch3.0/eNpHR3.0) optogenetic tools exerted qualitatively similar polarization on the bystander neuron membrane regardless of the nature of the transported ion (although with much slower kinetics than directly excited cells) and that minimal bulk pH changes were detected in the extracellular space, supports the conclusion that the coupling is predominantly ephaptic in nature. The fact that similar results were seen with electrically-induced changes in neuronal activity suggested that emergence of bystander currents is a general neuronal phenomenon, independent of the conducted/pumped ion species and now addressable experimentally with substantially improved specificity and speed. These findings may help guide inhibitory opsin selection for certain kinds of experiments, alongside recent investigations of presynaptic terminal inhibtion^58^. As is well-understood in the field^59^,^60^,^61^,^62^,^63^,^64^,^65^,^66^, for rigorous optogenetic or other modulations it has always been best practice 1) to match experimentally-delivered changes in activity to naturally-occurring patterns (for example, as measured by electrical or optical recordings of native activity^61^,^62^; 2) where indicated, to use moderate or asynchronous stimuli as dictated by the question at hand^63^,^64^ and 3) to carry out control experiments in which distinct neural populations or projections are recruited or inhibited so that specificity for the targeted population is established^65^. The results presented here support continued consideration of these long-standing principles for rigorous optogenetic (or other neuromodulatory) experimental design; the use of optogenetics in particular affords unique advantages in addressing these issues (compared with other interventional methods) in allowing readily-tunable intensity, sparsity, asynchrony, and brevity or chronicity of delivered modulations^66^.

Passive electrical charging of membrane capacitance by changes in the surrounding electric field could in part explain the observed phenomena. This “ephaptic” coupling has been described previously^67^,^68^,^69^, although usually at faster timescales. The slower timescale observed here (seconds-minutes) is perhaps consistent with a changing electric field convolved with the time course of activation of multiple ionic currents - both optogenetic as well as secondarily-activated currents such as ASIC currents. In the case of ChR2-induced bystander currents, metabotropic glutamate receptors (which were not blocked in these experiments) or other ion exchangers (e.g. Na^+^-H^+^ exchangers, which can also be blocked by amiloride), may also contribute to bystander depolarization. Other extracellular ions may play a role–for example K^+^, which will accumulate in extracellular space in the case of native activity or ChR2-induced depolarization, or diminish in the case of native or experimentally-induced reductions in activity (e.g. pharmacological or optogenetic hyperpolarization^3^,^49^,^70^). Equally, glial cells could contribute by mediating changes in extracellular pH through sodium-bicarbonate co-transport^49^,^50^,^71^. We note that a recent study in which the light-activated proton pump ArchT was expressed in glia^36^ found that light-induced proton extrusion from glia was sufficient to modulate ASIC activity on neighboring neurons, suggesting that glial-neuron interactions and neuron-neuron interactions may be differentially gated in the extracellular compartment.

By combining novel optogenetic engineering approaches, electrophysiology, pH-measurements and pharmacology this study highlights the intricate and complex relationship between neurons and their extracellular environment, and opens avenues for exploration of extracellular regulation of neural activity. We introduce a modular optogenetic approach, which can be used to study physiological interactions between genetically-specified cell populations and their direct microenvironment with high temporal and genetic precision. Cell-type specific activation of channels with high permeability for single ions also poses numerous advantages for investigation of downstream intracellular signaling. In this example, ASIC2a provides high sodium selectivity, however this module could be ‘switched out’ for a different proton-sensitive ion conductance, such as a channel conducting K^+^, Ca^2+^ or Cl^−^, allowing a breadth of different manipulations. Finally, the efficiency of ASIC activation by highly localized proton microdomains at the membrane surface raises new questions regarding the influence of different spatial compartments of the extracellular space on dendritic and axonal physiological processes, such as synaptic integration, action potential propagation and spatial regulation of tissue excitability.

## Methods

### Two-component optogenetics experiments

Construct design and expression in *Xenopus laevis* oocytes: The coding sequences for rat ASICs (in pRSSP6009) were provided by Stefan Gründer (Aachen). The pRSSP6009 plasmid coding for ASICs and the pGEM plasmid coding for Coccomyxa subellipsoidea C-169 Rhodopsin CsR_T46N_ were linearized by MulI site in pRSSP 6009 and by NheI in pGEM. After transcription into RNA using T7 (pGEM) or SP6 (pRSSP6009) mMessage mMachine Kit (Ambion Inc, Texas, USA) 32 ng of capped RNA encoding CsR pump and one type of ASIC were co-injected into *Xenopus leavis* oocytes with a molar ratio of 1:1 pump: channel for ASIC1 and ASIC2a and a molar ratio of 1:2 for ASIC3. Oocytes were incubated for 3 days at 18 °C in ORI solution with 1 μM all-trans retinal^72^.

#### Two-electrode voltage clamp measurements

TEVC measurements on *X. laevis* oocytes were performed using a GeneClamp 500 amplifier (Axon Instruments, Union City). Data acquisition, buffer exchange and light triggering were controlled with pClamp software via a Digidata 1322A interface (Molecular Devices, Sunnyvale). The light supplied by a 75 W Xenon lamp (Jena-Instruments, Jena, Germany) was passed through a K55 filter (Balzers, Liechtenstein) and applied to the oocytes using a light guide (diameter of 2 mm). The light intensity was 2.96 mW mm^−2^ at the surface of the oocyte. The bulk buffer (chamber volume 300 μl) was continuously perfused at a flow rate of 1.8 ± 0.2 ml min^−1^. Data was acquired at 1 kHz and filtered at 0.5 kHz. If not otherwise specified the extracellular buffer was composed of 100 mM NaCl, 1 mM KCl, 1 mM MgCl_2_, 0.1 mM CaCl_2_ and 0.1 to 5 mM MOPS at pH 7.5. For pH titration, buffer solutions were adjusted with 5 mM MOPS/MES/Citrate over the range of pH 8.0 to 4.0, and were subsequently compared with photocurrents measured at 0.1 mM MOPS at pH 7.5

#### Construct design and expression in HEK293 cells

For the design of bHK_pHluorin, bHK_pHluorin_CsR_T46N and CsR_T46N_eCFP_P2A_bHK_pHluorin the first 105 N-erminal amino acids of rat gastric H,K-ATPase ß-subunit bHK (NM_012510.2) were fused as a membrane anchor by a short 12 amino acid linker to superecliptic pHluorin (AY533296.1). bHK_pHluorin was then ether directly fused to the N-terminus of Coccomyxa subellipsoidea C-169 Rhodopsin CsR_T46N_ by a 5 amino acid linker or fused to CsR_T46N__eCFP by a P2A viral cleavage site or directly cloned into a pECFP-C1 backbone. All constructs were expressed under the control of a CMV-promotor. HEK293 cells were cultured in Dulbecco’s minimal essential medium supplemented with 10% (v/v) fetal bovine serum, 2 mM glutamine (Biochrome, Berlin, Germany), 1 *μ*M all trans-retinal, 175 *μ*M penicillin, 68 *μ*M streptomycin, 120 *μ*M blasticidine, and 175 *μ*M zeocine. Cells were seeded on coverslips at a concentration of 0.75 × 10^5^ cells/ml and transiently transfection using Fugene HD (Roche, Mannheim, Germany) was performed 28–36 h before measurement.

#### pH imaging in HEK293 cells

For fluorescence excitation a light guide of the Polychrome V unit (TILL Photonics, Planegg, Germany) was mounted at the epi-illumination port of an Olympus iX70 inverted microscope. For CsR activation a Xenon lamp (Jena-Instruments, Jena, Germany) was used and light color was selected by a bandpass filter centered at 560 nm with a FWHM of 10 nm. Light was coupled into a 3 mm light guide also mounted into the epi- illumination path of the microscope and combined with the beam from the polychrome unit via a 70% R/30% T beam splitter. Combined pHluorin and CsR excitation light was guided to the objective via a dualband dichroic mirror (FF493/574, AHF- Analysetechnik, Tübingen, Germany). This allowed us to excite pHluorin at 480 nm at a light intensity of 0.34 mW × mm^−2^ and monitor fluorescence between 500 and 530 nm recorded by a CCD Imago camera (TILL Photonics, Planegg, Germany). Recordings were performed at the following ion composition [in mM]: 140 NaCl, 2 MgCl_2_, 2 CaCl_2_, 1 KCl, 0.1/1/10 HEPES. pH was adjusted to pH 7.5 with NaOH/HCl. pHluorin fluorescence was recorded at an exposure time of 20 ms and a sampling rate of 1 Hz. CsR was excited at 560 nm for 15 s at a light intensity of 1.89 mW × mm^−2^. After each measurement fluorescence of surface exposed pHluorin was calibrated by subsequent buffer exchanges to pH 9 for maximal fluorescence and pH 5 for quenching of extracellular pHluorin. Cells showing more than 20% of maximum pHluorin fluorescence after quenching at pH 5 due to intracellular pHluorin were excluded from further analysis. Separately subcellular localization of bHK_pHluorin and bHK_pHluorin_CsR_T46N in HEK-293 cells was monitored 2 day after transfection with a confocal FluoView 1000 microscopy system (Olympus). Cell membrane was labeled by 2 μM Octadecyl Rhodamin B Chloride (R18) (Molecular Probes). Pictures were taken on a confocal LSM IX81 equipped with a 60 × 1.2 WaterUplanSApo objective (Olympus). A 488 nm laser diode operating at 1.0% and and a 559 nm laser diode operating at 5% was used to excite pHluorin and R-18 respectively. Fluorescence emission was detected at 510 nm and 591 nm using a photomultiplier-tube.

#### Construct design for hippocampal neurons

The protein sequence of rat ASIC2a (Genbank accession number AX286636) was human codon optimized and synthesized by Genscript. eArch3.0 and ASIC-YFP fusions were cloned into an AAV2 backbone either under a CaMKIIα or human synapsin promoter. The trafficking signal (TS) and endoplasmic reticulum export signal (ER) sequences were appropriately added to enhance membrane trafficking. All maps and sequence details are on the website: www.optogenetics.org.

#### Hippocampal neuron culture and calcium phosphate transfection

Experiments were performed as described previously^12^. Primary cultured hippocampal neurons were prepared from P0 Sprague-Dawley rat pups (Charles River). CA1 and CA3 were isolated, digested with 0.4 mg ml^−1^ papain (Worthington), and plated onto glass coverslips precoated with 1:30 Matrigel (Becton Dickinson Labware). Cultures were maintained in a 5% CO_2_ humid incubator with Neurobasal-A medium (Invitrogen) containing 1.25% FBS (HyClone), 4% B-27 supplement (Gibco), 2 mM Glutamax (Gibco) and 2 mg ml^−1^ fluorodeoxyuridine (FUDR) (Sigma), and grown on coverslips in a 24-well plate at a density of 65,000 cells per well. For each well, a DNA-CaCl_2_ mix was prepared with 2 μg DNA (Qiagen endotoxin-free preparation) and 1.875 μl 2 M CaCl_2_ (final Ca^2+^ concentration 250 mM) in 15 μl H_2_O. To DNA-CaCl_2_ we added 15 μl of 2 × HEPES-buffered saline (pH 7.05). After 20 min at room temperature (20–22 °C), the mix was added dropwise into each well (from which the growth medium had been removed and replaced with pre-warmed minimal essential medium, MEM) and transfection proceeded for 45–60 min at 37 °C, after which each well was washed with 3 × 1 ml warm MEM before the original growth medium was returned.

#### Electrophysiological recordings in cultured hippocampal neurons

Whole cell patch clamp recordings were performed on cultured hippocampal neurons 4–8 days post-transfection on an upright Leica DM-LFSA microscope as described previously^12^. Cells were continuously perfused in standard extracellular Tyrode’s solution (NaCl: 125 mM, KCl 2 mM, CaCl_2_ 2 mM, MgCl_2_ 2 mM, glucose 30 mM, HEPES 25 mM, titrated to pH 7.3–7.4 with NaOH, 320 mOsm) or in low HEPES Tyrode’s solution (NaCl: 125 mM, KCl 2 mM, CaCl_2_ 2 mM, MgCl_2_ 2 mM, glucose 55 mM, HEPES 0.1 mM, titrated to pH 7.3–7.4, 320 mOsm) at a rate of 1–2 ml min^−1^, in the presence of synaptic transmission blockers 6-cyano-7-nitroquinoxaline-2,3-dione (CNQX), D(-)-2-amino-5-phosphonovaleric acid (APV) and gabazine (25 μM; Tocris Bioscience). Patch pipette borosilicate glass electrodes (Sutter Instruments) with tip resistance of 3–6 MΩ were filled with a potassium gluconate intracellular solution (K-gluconate 130 mM, KCl 10 mM, HEPES 10 mM, EGTA 10 mM, MgCl_2_ 2 mM, titrated to pH 7.3 with KOH, 300 mOsm). Data acquisition, current and light manipulations were controlled using pClamp (Axon Instruments) via a Digidata 1440A interface and analyzed using ClampFit software (Axon Instruments). Data were acquired at 10 kHz. Cells were held at −70 mV for all voltage-clamp experiments. Resting membrane potentials were corrected offline for an estimated liquid junction potential of 16 mV. Full-field illumination for activation of optogenetic tools was delivered by a 300 W DG-4 lamp (Sutter instruments) via a 40X, 0.8 numerical aperture (NA) water-immersion objective. The light was first passed through a 560/25 nm filter within a Lambda 10–3 filter wheel (Sutter Instruments). Light power density was ~5 mW mm^−2^ for all experiments. All experiments were performed at room temperature (24–25 °C). Data were collected across multiple transfected cultures.

#### Assessment of modulation of calcium-dependent pathways in Champ-expressing neurons

Plates of cultured hippocampal neurons were simultaneously transfected with Champ3.0, eYFP only, or nothing. On the experimental day (4–6 days post-transfection), the plates were prepared by incubation in standard HEPES Tyrode’s solution (described above) containing ionotropic synaptic blockers (APV, CNQX and gabazine, 50 μM) and a voltage-gated Ca^2+^ channel blocker, nifedipine (10 μM), at room temperature for 20 minutes. Plates were then individually loaded onto the patch-clamp rig and continually perfused with standard Tyrode’s solution containing gabazine and CNQX (50 μM) but no APV or nifedipine. The plates containing non-transfected cells were perfused with a high potassium (90 mM) containing-Tyrode’s solution, with concurrent reduction in sodium concentration (39 mM) to maintain constant osmolarity, and the same synaptic blockers as described above. All plates were exposed to 560 nm light at ~5 mW/mm^2^ from a 40x/0.8 NA water-immersion objective for 5 minutes, in epochs of 30 s on/30 s off. Following light stimulation, cells were immediately fixed in PFA for detecting activation of CREB and MAPK, or were returned to Tyrode’s solution and kept in the dark at 32 °C for a further 90 minutes prior to fixation for detection of cFos activation. Two replicate plates were used for each antibody for each condition: Champ3.0, YFP control or high K^+^ control (a total of 18 plates). After 3 hours of fixation, plates were washed in 1X phosphate buffered saline and incubated in blocking solution for 3 hours. After blocking, plates were incubated in primary antibodies for 24 hours. The following primary antibodies were used: monoclonal anti-MAPK (activated, diphosphorylated ERK1&2, Sigma Aldrich M8159, 1:500 dilution^73^,^74^, monoclonal anti-CREB (phospho S133, Abcam ab32096, 1:500 dilution)^75^, polyclonal anti-cFos (Abcam ab53036, 1:500 dilution)^76^. Following a second wash, Alexa Fluor® 647 secondary antibody (Abcam ab150079, 1:500 dilution) was applied in 2% NDS for 1 hour at room temperature for 3 hours followed by DAPI (1:50,000) for 30 mins, then mounted, and coverslipped with PVA-DABCO (Sigma). Images were obtained on a Leica confocal microscope (DM600B) at 1024 × 1024 pixel resolution using 5x and 10x dry objectives and 20x, 40x and 63x oil objectives. All images were taken under matched illumination and gain for the calcium-activated protein channel. Mean fluorescence intensity was calculated for a sample of ~20 cells per condition. For Champ3.0 and YFP cells, only YFP-expressing cells were included in the analysis.

#### Confocal images of cultured neurons

All other confocal images of TCO constructs in neurons were obtained by staining glass coverslips of transfected neurons with DAPI (1:50,000) which were then imaged using a Leica confocal microscope (DM600B) at 1,025 × 1,024 pixel resolution, at 40X magnification, 1.25 NA (oil). Excitation/emission wavelengths for eYFP were 488 nm/500–545 nm.

### Bystander experiments

All experiments were conducted under protocols approved by the Stanford Administrative Panel on Laboratory Animal Care (APLAC) and were performed in accordance with APLAC guidelines. Animals were housed on a 12 hour light-dark cycle (not reversed) in cages housing up to 5 animals.

#### Stereotactic injections

For expression of ChR2_(H134R)_, eArch3.0 or eNpHR3.0 in CamKII-positive neurons, adeno-associated virus (AAV) serotype 2/5 was produced by the University of North Carolina Chapel Hill Vector Core at a genomic titer of ~4–16 × 10^12^ pfu mL^−1^. We stereotactically injected 1 μL of virus at two sites unilaterally into the CA1 region of the hippocampus of 3–4 week-old mice (C57BL/6J, Jackson Laboratories, male and female). Coordinates for all animals at injection site #1 were −2.2 anteroposterior, 1.5 mediolateral (left side) and 1.3 dorsoventral (in mm from bregma) and for injection site #2 were −1.7 anteroposterior, 1.25 mediolateral (left side) and 1.5 dorsoventral (in mm from bregma). For cortical bystander experiments, *Thy1::ChR2* (line 18, C57BL/6J) mice (Arenkiel *et al.*^47^) were used (bred in-house).

#### Acute slice electrophysiology recordings

Acute brain slices were prepared from mice at 4–8 weeks post virus injection, or at 4 weeks of age for transgenic mice. After lethal anesthesia, transcardial perfusion was performed prior to decapitation, followed by rapid brain extraction and submersion of the brain in ice-cold sucrose-based slicing solution (234 mM sucrose, 26 mM NaHCO_3_, 11 mM glucose, 10 mM MgSO_4_.7H_2_0, 2.5 KCl, 1.25 mM NaH_2_PO_4_.H_2_O, 0.5 mM CaCl_2_.2H_2_0). 300 μm thick slices of hippocampus were cut on a Leica vibratome (Leica VT1000S). After cutting, slices were submerged in a hypertonic recovery solution (artificial cerebrospinal fluid (ACSF) at an 8% increased concentration) at 33 °C for 15 mins before being transferred to standard ACSF (123 mM NaCl, 26 mM NaHCO_3_, 11 mM glucose, 3 mM KCl, 2 mM CaCl_2_.2H_2_0, 1.25 mM NaH_2_PO_4_.H_2_O, 1 mM MgCl_2_.6H_2_O) for a further 45 mins at 33 °C, at which point they were transferred to room temperature.

Whole cell patch clamp recordings on cortical and hippocampus neurons were performed on an upright Leica DM-LFSA microscope. Slices were continually perfused in warmed (33 °C) ACSF at a rate of 7 ml min^−1^. Patching was performed in the presence of synaptic transmission blockers 6-cyano-7-nitroquinoxaline-2,3-dione (CNQX, 50 μM) and D(–)-2-amino-5-phosphonovaleric acid (APV, 25 μM) and gabazine (25 μM) (Tocris Bioscience) except for during testing of electrically-evoked synaptic responses. For pharmacological experiments, amiloride (500 μM, Tocris Bioscience, 15–20 mins perfusion) or acetazolamide (500 μM, Santa Cruz Biotech, 5 to 15 mins perfusion) were added to the ACSF.

Borosilicate glass (Sutter Instruments) pipette resistances were pulled to 3–6 MΩ and filled with potassium gluconate intracellular solution (130 mM KGluconate, 10 mM KCl, 10 mM HEPES, 10 mM EGTA, 2 mM MgCl_2_, pH adjusted with KOH to 7.3). Voltage and current clamp electrophysiological recordings and manipulations were performed using pClamp (Axon Instruments). Data were acquired at 10 kHz. Cells were held at −70 mV for all experiments. Cells with leak current greater than −250 pA or pipette resistances greater than 35 MΩ were excluded. Light (full-field illumination) was emitted from a 300 W DG-4 lamp (Sutter Instruments, Novato, CA, USA) fitted with a Lambda 10–3 filter wheel (Sutter Instruments) with a 10-position wheel for filters of different wavelengths, or external filters (wavelength in nm/bandwidth in nm: 470/20; 560/25; 590/20). Light pulses were delivered through a 40x, 0.8 NA water-immersion objective at 4–7 mW/mm^2^ light power density.

Extracellular electrical stimulation was performed using a concentric bipolar electrode of platinum iridium (FHC, Bowdoin, ME, USA) or tungsten (World Precision Instruments, Sarasota, FL, USA). Electrical pulses were delivered using a stimulus isolator (ISO-Flex, A.M.P.I) controlled by pClamp to deliver 200 μs square pulses at intensities ranging from 50 μA to 5 mA and frequencies of 5–10 Hz.

#### pH recordings

Extracellular pH measurements from acute slices were obtained using a solid state metal wire oxide pH sensor (100 μm diameter, Beetrode NMPH1, World Precision Instruments, Inc.) with a liquid junction reference electrode (DRIREF-450, World Precision Instruments, Inc.) connected to a Jenco pH meter (JE671P). Calibration of the sensor was performed immediately prior to the start of each experiment. pH recordings were allowed to stabilize before measurements began and recordings in which baseline drift exceeded more than four standard deviations from the mean were excluded from the analysis. pH measurements were sampled every 15 s, commencing with a two minute baseline period, followed by 3 minutes of continuous light stimulation (~5 mW/mm^2^ from a 40x, 0.8 NA water-immersion objective positioned above the recording site, followed by a 3 minute “light-off” recovery period. Acute slices were prepared as described above and the experiments were conducted in the same solutions as used for electrophysiological recordings.

#### Immunohistochemistry

For identification of bystanders in slice preparations, 0.3% biocytin was added to the intracellular pipette solution and following recording slices were fixed in 4% paraformaldehyde perfusion fix solution (Electron Microscopy Services, Hatfield, PA, USA) for 24 hours then transferred to 1X phophate buffered saline (Gibco, Life Technologies). Biocytin was stained with fluorescent streptavidin (Alexa Fluor® 546 conjugate, Invitrogen S-11225, over 3 hours). For YFP staining, slices were incubated in anti-GFP primary antibody (Invitrogen G10362, 1:500 dilution)^77^ for 24 hours. Cy5 secondary antibody (Jackson Laboratories, West Grove, PA, 111-175-144, 1:500 dilution) was applied in 2% NDS for 3 hours at room temperature followed by DAPI (1:50,000) for 30 mins, then mounted, and cover-slipped with Fluoromount (Sigma). Images were obtained on a Leica confocal microscope (DM600B) at 1024 × 1024 pixel resolution using 5x and 10x dry objectives and 20x, 40x and 63x oil objectives.

To detect the presence of ASIC channels in hippocampus, mice were deeply anaesthetized with isoflurane and Beuthanasia-D and were transcardially perfused in cold 4% paraformaldehyde perfusion fix solution (Electron Microscopy Services, Hatfield, PA, USA). Brains were extracted and kept in the fixation solution for 24 hours at 4 °C and then transferred to 30% sucrose in PBS to equilibrate for 3 days at 4 °C. 40 μm slices were cut on a freezing microtome and stored in cryoprotectant at 4 °C. Immunotaining was performed as described above, using the following primary antibodies: anti-ASIC1 (rabbit polyclonal Ab, Alomone labs, ASC-014, 1:200 dilution), anti-ASIC2a (rabbit polyclonal Ab, Alomone labs, ASC-012, 1:200 dilution)^78^,^79^.

#### Data analysis

Analysis of all of physiological results was performed using Clampfit software (Axon Instruments). Pipette (access) resistance (R_a_) and membrane resistance (R_m_) were monitored at 5 minute intervals to ensure stability of the recording and data was only included when leak current was less than 250 pA and R_a_ less than 35 MΩ. Reversal potentials were corrected offline for an estimated liquid junction potential of 14 mV. Data were acquired at 10 kHz. Voltage clamp data for bystander currents were filtered at 100 Hz for visualization. Statistical analysis was performed using GraphPad Prism 6.0 for Mac OS X and Microsoft Excel. For comparisons between YFP controls and opsin or electrical stimulation groups, non-parametric unpaired two-tailed Mann Whitney tests were used to compare rank sums between groups, to avoid the assumption of a Gaussian distribution. For comparison of the functional impact of light on bystander neuron spiking over successive light-on vs light-off epochs, a repeated measures one-way ANOVA was performed for each opsin, with correction for multiple comparisons using Tukey’s multiple comparison test. For analyzing the effect of acetazolamide treatment on ChR2 and eArch3.0-induced bystander responses, paired two-tailed t-tests were used. For analyzing the effect amiloride treatment on ChR2-induced bystander responses, a two-way ANOVA with correction for multiple comparisons using Dunnet’s multiple comparison test. Significance thresholds were set at p < 0.05 (*), p < 0.01 (**), p < 0.001 (***) and p < 0.0001 (****).

## Additional Information

**How to cite this article**: Ferenczi, E. A. *et al.* Optogenetic approaches addressing extracellular modulation of neural excitability. *Sci. Rep.* **6**, 23947; doi: 10.1038/srep23947 (2016).

## Supplementary Material

Supplementary Information
