# Supplementary material for: Optogenetic approaches addressing extracellular modulation of neural excitability
Source: Sci Rep. 2016 Apr 5;6:23947. doi: 10.1038/srep23947 (PMC4820717; doi:10.1038/srep23947)
Supplement: Supplementary Information [file srep23947-s1.pdf]

Supplementary Information

Optogenetic approaches addressing extracellular modulation of neural excitability

*Emily Ferenczi[3,4], *Johannes Vierock[1], Kyoko Atsuta-Tsunoda[1], Satoshi P. Tsunoda[1], Charu Ramakrishnan[3], Chris Gorini[3], Kimberly Thompson[3], Soo Yeun Lee[3], Andre Berndt[3], Chelsey Perry[3], Sonja Minniberger[1], Arend Vogt[1], Joanna Mattis[3, 4], Rohit Prakash[3,4], Scott Delp[3], #Karl Deisseroth[2,3,5] , #Peter Hegemann[1]

[1]Institute of Biology, Experimental Biophysics, Humboldt-Universität zu Berlin, Berlin, Germany

[2]HHMI, [3]Bioengineering, [4]Neurosciences, [5]Psychiatry & Behavioral Sci., Stanford University, Stanford, CA, USA

* Equal contributors

# Corresponding authors: deissero@stanford.edu, hegemann@rz.hu-berlin.de

This document contains:

Supplementary Figure Legends 1-12

Supplementary Figs 1-12

Supplementary Figure 1: Photocurrents of *Coccomyxa subellipsoidea* rhodopsin (CsR)

a, b Light induced pump currents of CsR WT (A) and T46N (B) in Xenopus oocytes. The T46N mutant exhibits greater currents at negative membrane voltage compared to WT. **c**, Current - voltage relationships, *I*(*E*), (mean +/- SD, n = 6 (WT), n = 8 (T46N)) and **d**, action spectra with maxima at 545 nm. **e**, Comparison of CsR photocurrents (545 nm excitation) with those of bacteriorhodopsin (BR, 570 nm excitation) and Arch expressed under identical condition (mean +/- SD, n = 7 (CsR), n = 6 (BR), n = 8 (Arch)). Current amplitudes of CsR are on average 10 fold greater than those of BR.

Supplementary Figure 2: Characterization of CsR-ASIC1a with and without continuous perfusion in *Xenopus leavis* oocytes.

In "perfusion" conditions a measuring chamber of 300 µl was continuously perfused with 1.8 ± 0.2 ml/min of standard measuring buffer containing 100 mM NaCl, 1 mM KCl, 1 mM $MgCl_2$, 0.1 mM $CaCl_2$, 0.1 mM MOPS (pH 7.5). In measurements "without perfusion" the buffer supply was disconnected and the peristaltic pump switched off. **a**, Representative CsR_{T46N}-ASIC2a photocurrents at 40 s light pulses of 560 nm in a succession of conditions "without perfusion", with continuous "perfusion" and "without perfusion" at different voltages. Insets: Repetitive photocurrent "without perfusion" and with "continuous perfusion" at -40 mV. **b**, Comparison of normalized peak photocurrent with "perfusion" (blue, circles) and "without perfusion" (grey, squares). Filled squares describe the response to the first light pulse and empty squares to the third light pulse (see **a**) (mean +/- SD, n = 6). **c**, Normalized peak photocurrent at -40 mV in dependence on the initial CsR_{T46N} pump current in "perfusion" condition (blue circle) and "without perfusion" (squares, filled for 1st light pulse empty for 3rd light pulse, see **a** and **b**). All currents were normalized to ASIC2a current at pH4 and -40 mV (see Figure 5). **d**, CsR_{T46N}-ASIC2a on-kinetics quantified by the time to reach the half maximal photocurrent $t_{1/2,on}$ with perfusion (blue, circles) and without perfusion (grey, filled and empty squares, see **b** and **c**) (mean +/- SD, n = 6). **e**, CsR_{T46N}-ASIC2a off-kinetics quantified by the time to reduce the photocurrent by half after light switched off $t_{1/2,off}$ with perfusion (blue, circles) and without perfusion (grey, filled and empty squares, see **b** and **c**) (mean +/- SD, n = 6). Inset: Description of $t_{1/2,on}$ and $t_{1/2,off}$ (red) on a representative photocurrent at -40 mV.

Supplementary Figure 3: Recovery of ASIC2a/CsR in *Xenopys laevis* oocytes

a, Repetitive activation of Asic2a/CsR in Xenopus oocytes by light (solid green line) in 0.1 mM MOPS or by a pH jump to pH 4 (blue dashed line) in 5 mM MOPS/citrate buffer. **b**, Mono-exponential fitted recovery kinetics of the pH activated current (blue, mean +- SD, $F(3,4) = 52.64$, $p = 0.0013$, $n = 4$), initial outward directed photocurrent (green, mean +- SD, $F(3,3) = 10.02$, $p = 0.047$, $n = 6$), light-induced inward current (red, mean +- SD, $F(3,3) = 6.43$, $p = 0.082$, $n = 6$).

Supplementary Figure 4: Variable presence of the ASIC2a component in cultured hippocampal neurons

a, Example of an eArch-ASIC2a current when the ASIC2a component is small (upper trace) and example of an ASIC negative current, in which only the eArch (outward) component is present, with no inward ASIC2a component (lower trace). **b**, Magnitude of outward current components for ASIC negative cells (eArch component present only, n = 23) and ASIC positive cells (both outward (eArch) and inward (ASIC2a) components present, n = 21). **c**, Voltage clamp holding current for all ASIC negative (n = 23) and ASIC positive (n = 21) cells. **d**, Resting membrane potential for all ASIC negative (n = 11) and ASIC positive (n = 13) cells. **e**, Pipette (access) resistance for all ASIC negative (n = 23) and ASIC positive (n = 21) cells. **f**, Membrane resistance for all ASIC negative (n = 23) and ASIC positive (n = 21) cells.

Supplementary Figure 5: Champ currents, kinetics and changes in membrane potential in response to light pulses of increasing duration

a, Magnitude of outward and inward current components in response to 1 s (n = 21), 5 s (n = 14) and 15 s (n = 10) light pulses. **b**, Off-kinetics of Champ current in response to 1 s (n = 18), 5 s (n = 9) and 15 s (n = 7) light pulses. The off-response is fitted by an exponential with a fast and slow term. **c**, Magnitude of membrane hyperpolarization and depolarization in response to 1 s (n = 13), 5 s, (n = 10) and 15 s (n = 4) light pulses. **d**, Number of spikes evoked in response to 1 s (n = 17), 5 s (n = 15) and 15 s (n = 4) light pulses.

Supplementary Figure 6: Calcium-signaling pathways are not disrupted by Champ3.0 activation

Immunostaining of cultured hippocampal neurons for markers of activation of calcium-dependent signaling pathways under three different conditions: exposure to high-potassium (90 mM) containing Tyrode's solution (positive control), Champ3.0 expression and YFP expression (negative control) after 5 minutes (epochs of 30 s on/30 s off) of green (560 nm) light stimulation. Images show examples of expression patterns for each condition. Summary plots indicate mean cell fluorescence in the calcium-activated protein channel for each condition. Bars indicate mean and SEM, each data point indicates an individual cell. **a**, Expression of activated MAPK (diphosphorylated ERK1/2). One-way ANOVA: $F = 367.8$, $p < 0.0001$ (number of treatments = 3, number of cells = 60), Tukey's multiple comparison test shows significant differences between high K^+ and Champ3.0/YFP, but no difference between Champ3.0 and YFP. **b**, Expression of activated CREB (phosophorylation at serine 133). One-way ANOVA: $F = 523.5$, $p < 0.0001$ (number of treatments = 3, number of cells = 61), Tukey's multiple comparison test shows significant differences between high K^+ and Champ3.0/YFP, but no difference between Champ3.0 and YFP. **c**, Expression of cFos. One-way ANOVA: $F = 18.53$, $p < 0.0001$ (number of treatments = 3, number of cells = 57), Tukey's multiple comparison test shows significant differences between high K^+ and Champ3.0/YFP, but no difference between Champ3.0 and YFP.

Supplementary Figure 7: Measures of cell health across four different Champ constructs with increasing molecular separation between proton pump and ASIC

There were no significant differences in any cell health measures across the 5 groups Champ3.0 (n = 16), Champ2.0 (n = 17), Champ4.0 (n = 14), co-transfection of eArch3.0 (n = 9) and ASIC2a and eArch3.0 only (n = 9) (as determined by one-way ANOVA) **a**, Holding current ($F(4, 60) = 1.561$, $p > 0.05$) **b**, Membrane resistance ($F(4, 60) = 2.194$, $p > 0.05$) **c**, Pipette resistance ($F(4, 60) = 1.876$, $p > 0.05$) **d**, Resting membrane potential ($F(4, 39) = 2.269$, $p > 0.05$).

Supplementary Figure 8: Comparing direct photocurrents and bystander currents

a, Example photocurrent traces for AAV5-CamKII-ChR2 (blue), eArch3.0 (green) and eNpHR3.0 (amber) expressing neurons, superimposed on example bystander currents at the same timescale (these data are also shown in Fig 6e-f). The full scale bystander currents are shown in the dashed boxes below. **b-c**, Example traces illustrating the difference in on-kinetics between

ChR2 expressing-cell photocurrents and ChR2 bystander currents in response to 470 nm light pulses. **a**, A strongly-expressing ChR2-photocurrent compared to a bystander current (responses to 0.5 s and 15 s pulses respectively). Dashed box shows a zoom-in of the first 0.5 s of both traces. **b**, A weakly expressing ChR2-photocurrent compared to a bystander current (responses to a 15 s light pulse). Even a very weak photocurrent (~20 pA) has fast (~ 10-15 ms) on-kinetics, distinguishing it from a bystander current. **d**, Average steady state direct photocurrent magnitudes (in response to 1 s light) for opsin-expressing neurons present in same preparations as bystander neurons: ChR2 (n = 20, 470 nm), Thy1-ChR2 (n = 6, 470 nm), eArch3.0 (n = 14, 560 nm), eNpHR3.0 (n = 16, 590 nm). Bars indicate mean and SEM, filled circles indicate individual cell photocurrents.

Supplementary Figure 9: Membrane resistance of bystander neurons and I-V plots for bystander currents

a, Change in membrane resistance from baseline in response to 30 s pulse of light for ChR2 (470 nm, 24% decrease in membrane resistance, Mann Whitney test for ChR2 vs YFP: n = 8, p = 0.0008, sum of ranks: 42, 148, U = 6), eArch3.0 (560 nm, 9% increase in membrane resistance, Mann Whitney test for eArch3.0 vs YFP: n = 12, $p < 0.0001$, sum of ranks: 203, 73, U = 7), eNpHR3.0 (590nm, 2% increase in membrane resistance, Mann Whitney test for eNpHR3.0 vs YFP: n = 11, p = 0.0083, sum of ranks: 166, 87, U = 21) and YFP control bystanders (560 nm, 3% decrease in membrane resistance, n = 11). **b**, Current-voltage relationships for depolarizing ChR2 bystander currents (R^2 for slope (difference from 0) = 0.71, $p < 0.0001$, n = 5) and hyperpolarizing bystander currents (eArch3.0: R^2 for slope = 0.43, $p < 0.0001$, n = 12; eNpHR3.0: R^2 for slope = 0.26, p = 0.0001, n = 10). **c-e**, Cell health measures for bystander neurons: **c**, Leak current, **d**, pipette (access) resistance and **e**, membrane resistance for ChR2 (n = 12), eArch3.0 (n = 12), eNpHR3.0 (n = 14) and YFP (n = 10) bystander neurons. Lines indicate mean and SEM.

Supplementary Figure 10: Strong electrical stimulation yields ChR2 bystander-like effects

a, Electrical stimulation (e-stim) bystanders were generated by extracellular electrical stimulation of Schaffer collaterals to CA1 region. **b**, Confirmation of synaptic input to CA1 region by low amplitude electrical stimulation (stimulation parameters and epoch shown in red).

c,**d**, Examples of a bystander response to high amplitude electrical stimulation of Schaffer collaterals with a platinum-iridium concentric bipolar electrode (**c**)**,** and a tungsten concentric bipolar electrode (**d**) in the presence of synaptic transmission blockers (stimulation artifact during 15 s e-stim epoch, stimulation parameters and epoch shown in red). **e**, Mean bystander currents for electrical stimulation (mean +/- SEM = -11 +/- 4 pA, $p < 0.001$, $n = 7$, sum of ranks: 30 (E-stim), 123 (YFP), U = 2), 10 Hz ChR2 stimulation ($n = 11$, data shown in Fig 1g, sum of ranks: 66 (ChR2), 165 (YFP), U = 0) and YFP controls ($n = 10$, data shown in Fig 1e). Bars indicate mean values and error bars represent standard error of the mean (SEM). Individual data points indicate results from single cells. Statistical comparisons are between the test (e-stim and ChR2) groups and YFP controls using the Mann Whitney paired test (non-parametric).

Supplementary Figure 11: Cell health data for amiloride and acetazolamide pharmacological experiments

Cell health parameters for over the course of the amiloride experiment (n = 6-18 for amiloride-treated cells (magenta), n = 4-16 for untreated control cells (grey). Period of amiloride (500 μM) application is indicated by the lilac-shaded region. **a**, Pipette resistance, **b**, membrane resistance (this data, normalized to baseline, is shown in Figure 3h) **c**, holding current. The bystander current was elicited every 5 mins by a 15 s pulse of 470 nm light. Symbols and error bars indicate mean and SEM respectively. **d-f**, Cell health parameters for eArch3.0 and ChR2 bystander neurons pre- and post-acetazolamide (500 μM) administration. **d**, Pipette resistance, **e**, membrane resistance, **f**, voltage clamp holding current. Lines indicate data for individual cells.

Supplementary Figure 12: Summary of extracellular modulation of neural excitability on two spatial scales

Summary of the two phenomena described in this paper: short range paracrine effects of proton microdomains on ASICs at the extracellular membrane surface (a) and longer range ephaptic-like effects of hyperpolarizing (b) and depolarizing (c) optogenetic tools with some contributions from ASICs, most likely at synapses. The schematic also highlights the possible contributions of other, as yet unknown, contributory mechanisms which remain to be discovered.

Supplementary Figure 1

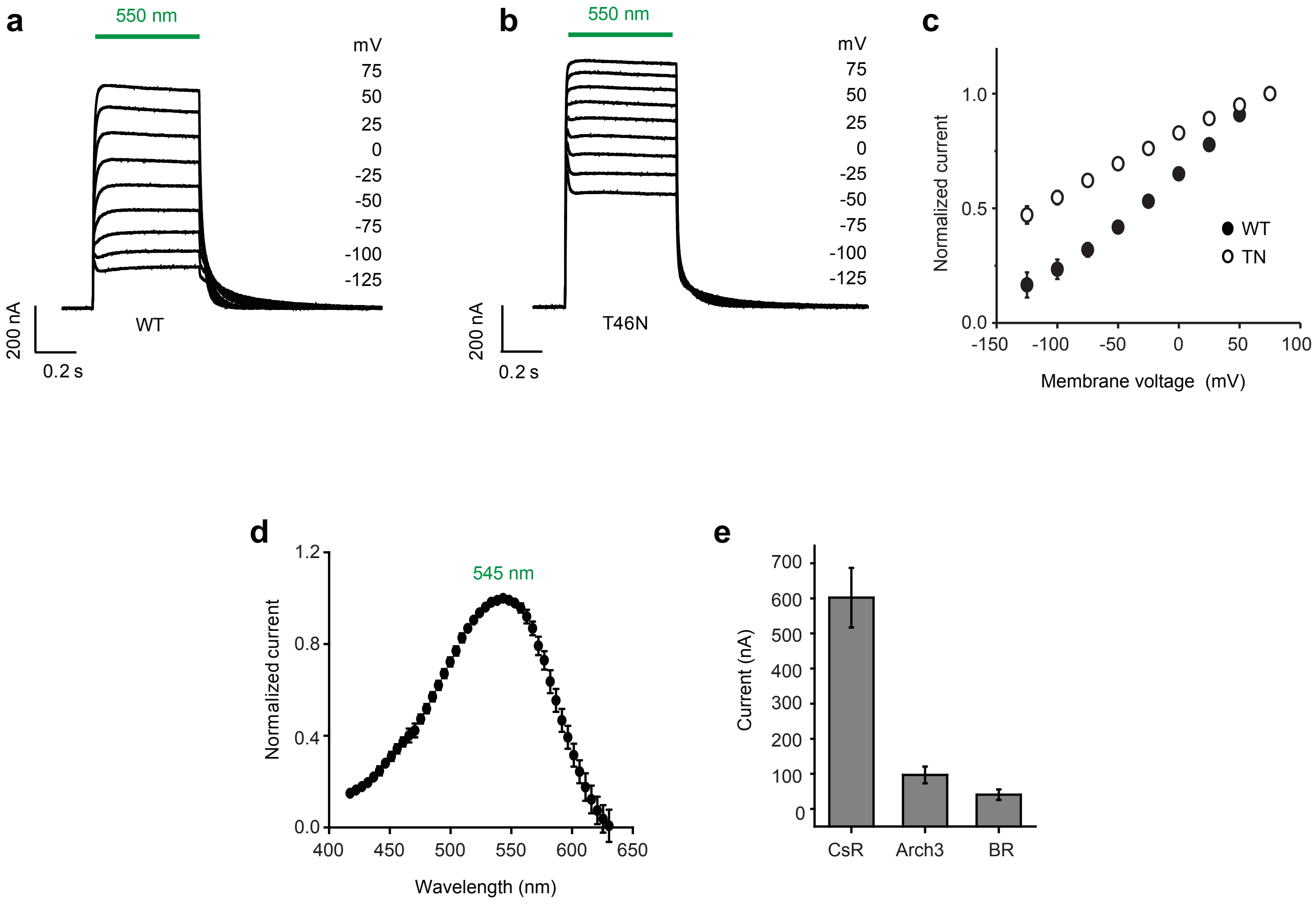

Supplementary Figure 2

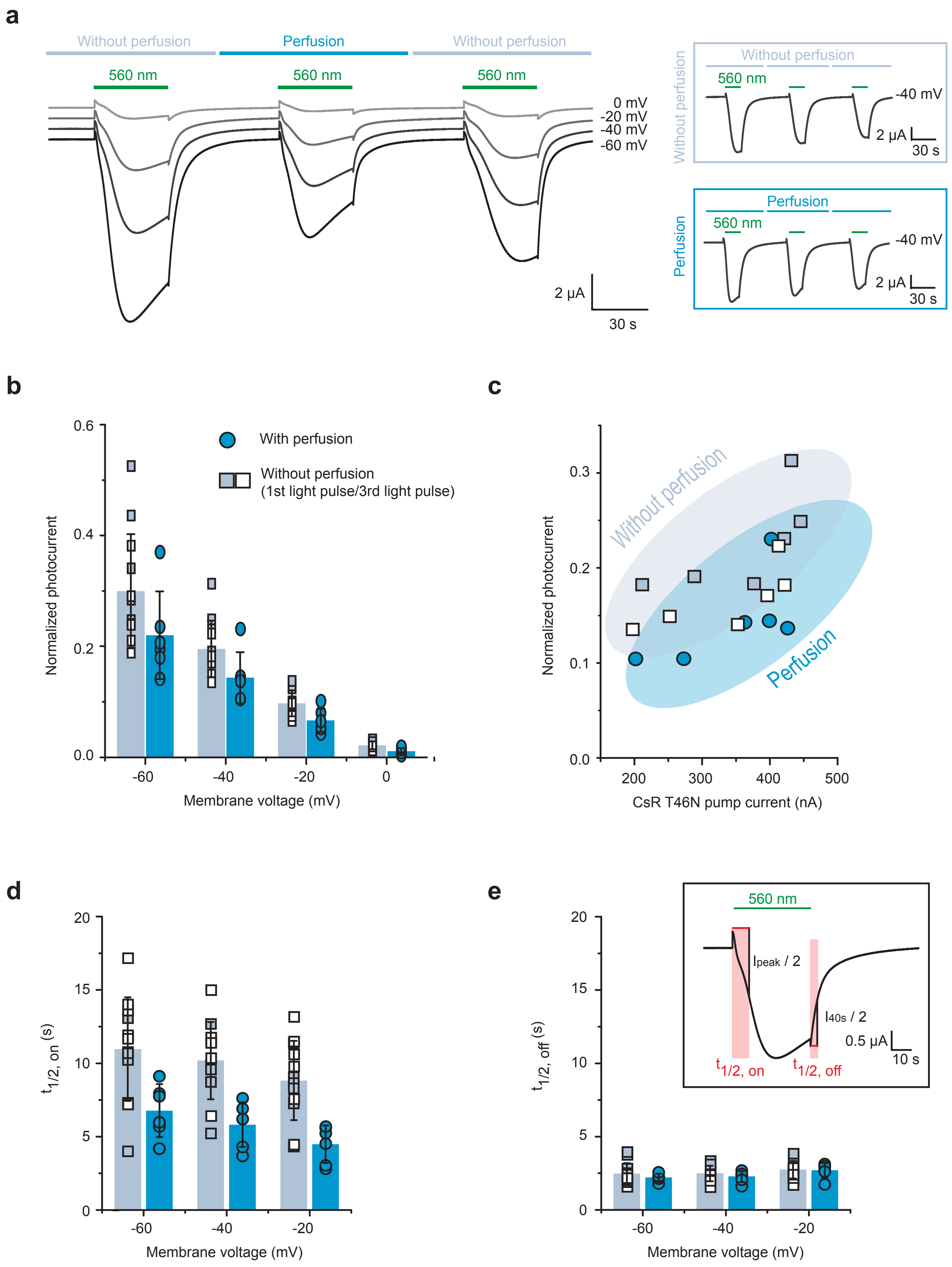

Supplementary Figure 3

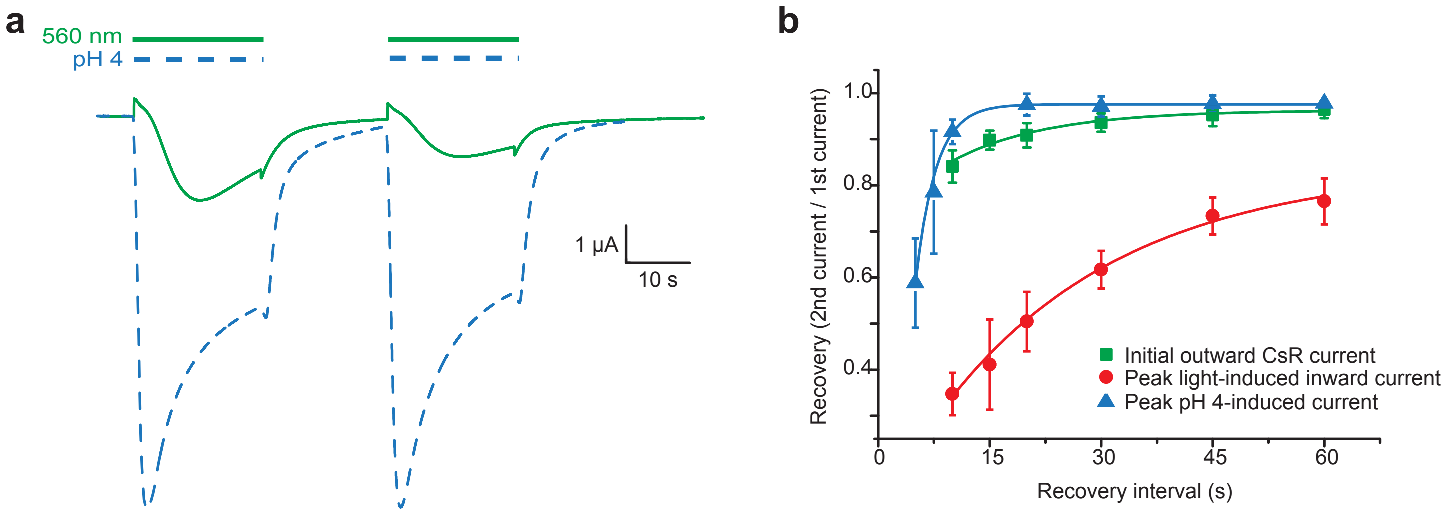

Supplementary Figure 4

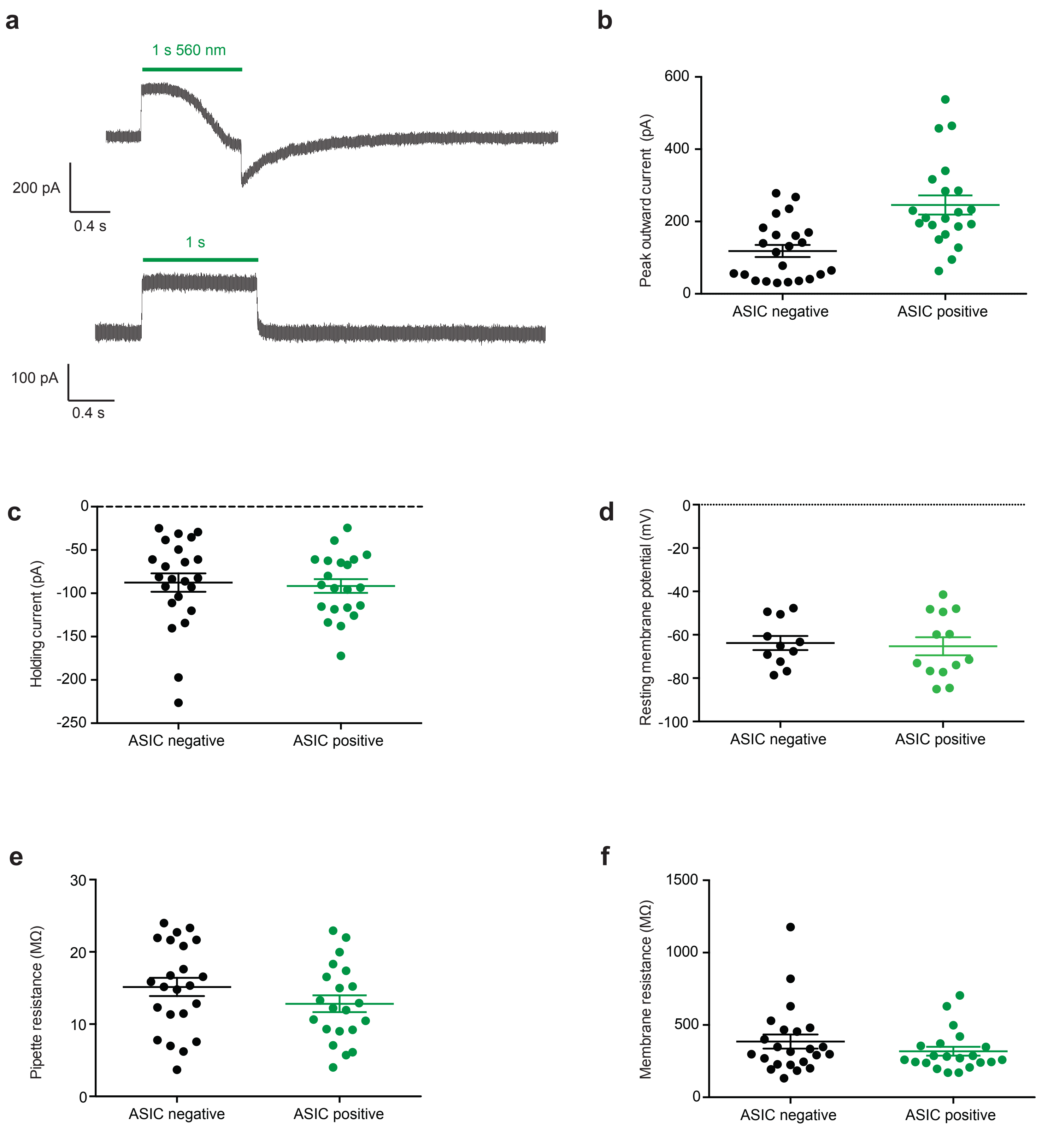

Supplementary Figure 5

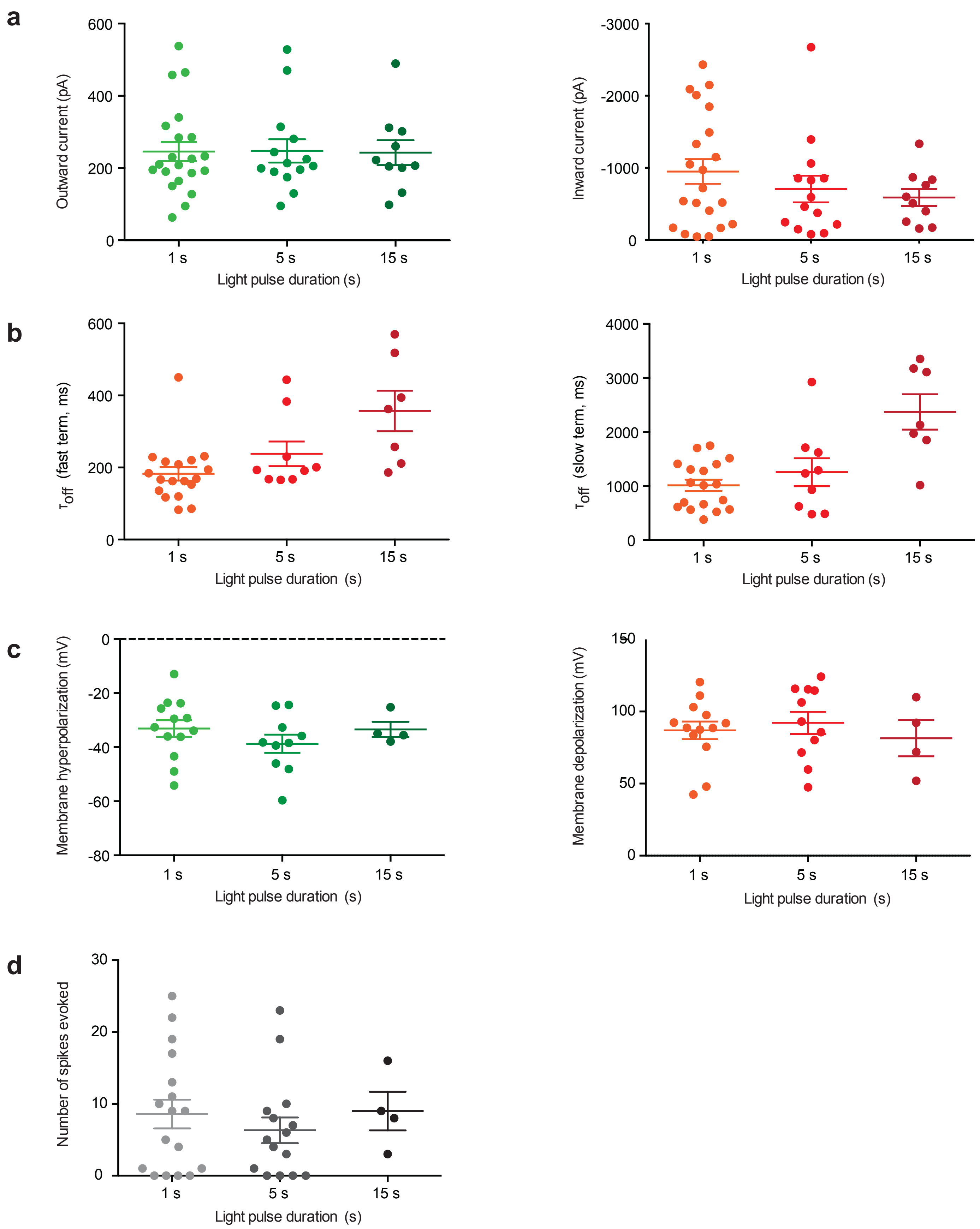

Supplementary Figure 6

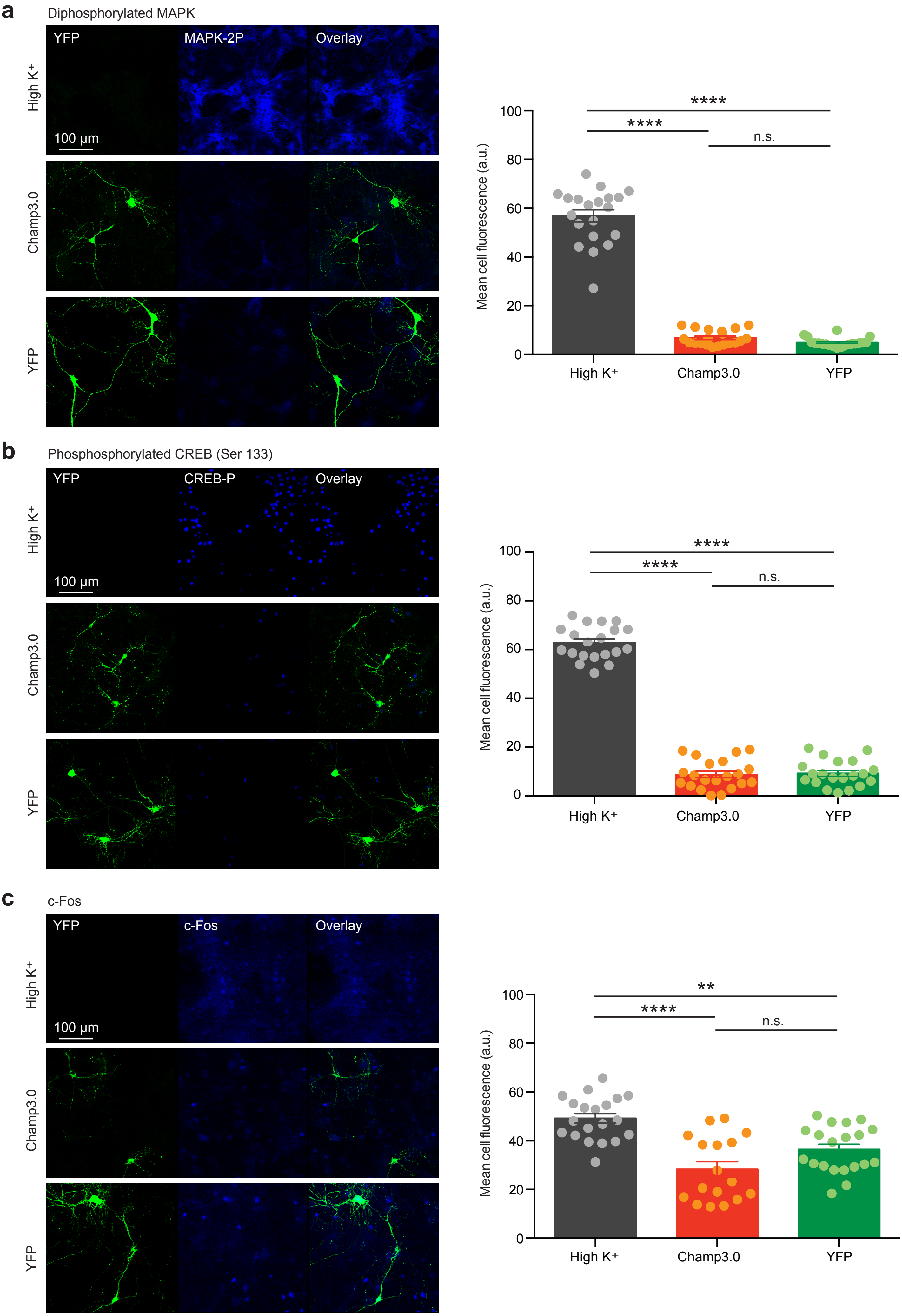

Supplementary Figure 7

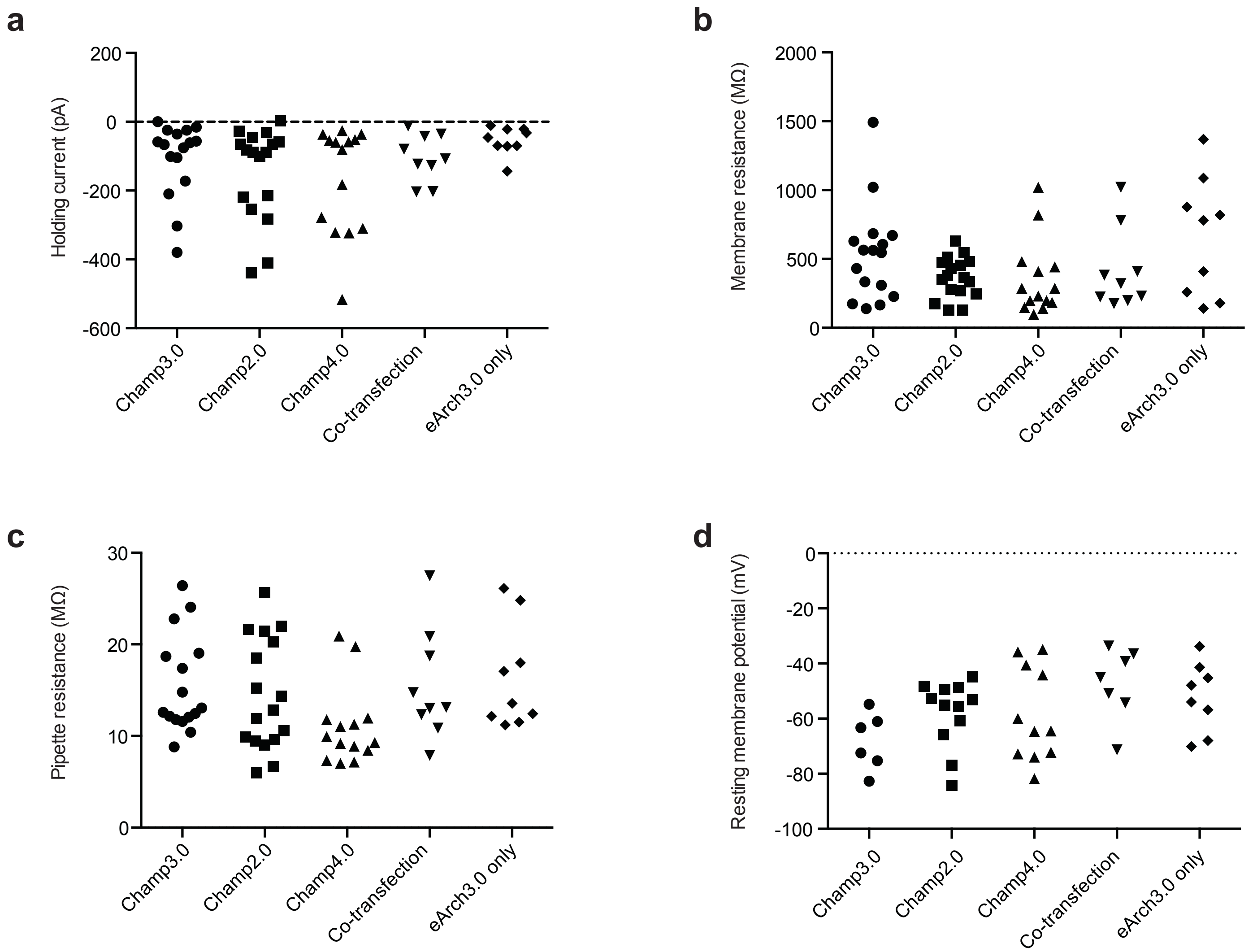

Supplementary Figure 8

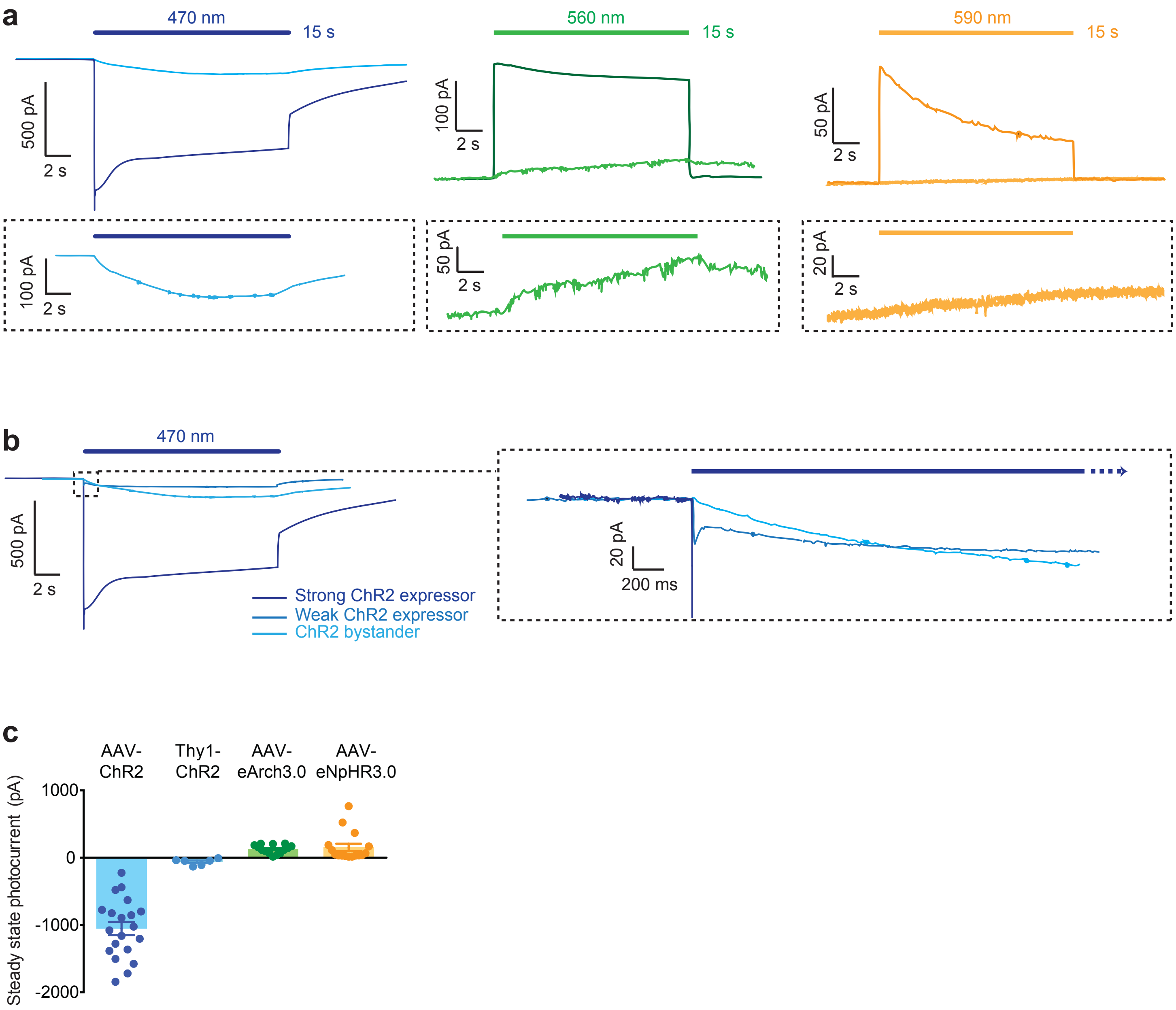

Supplementary Figure 9

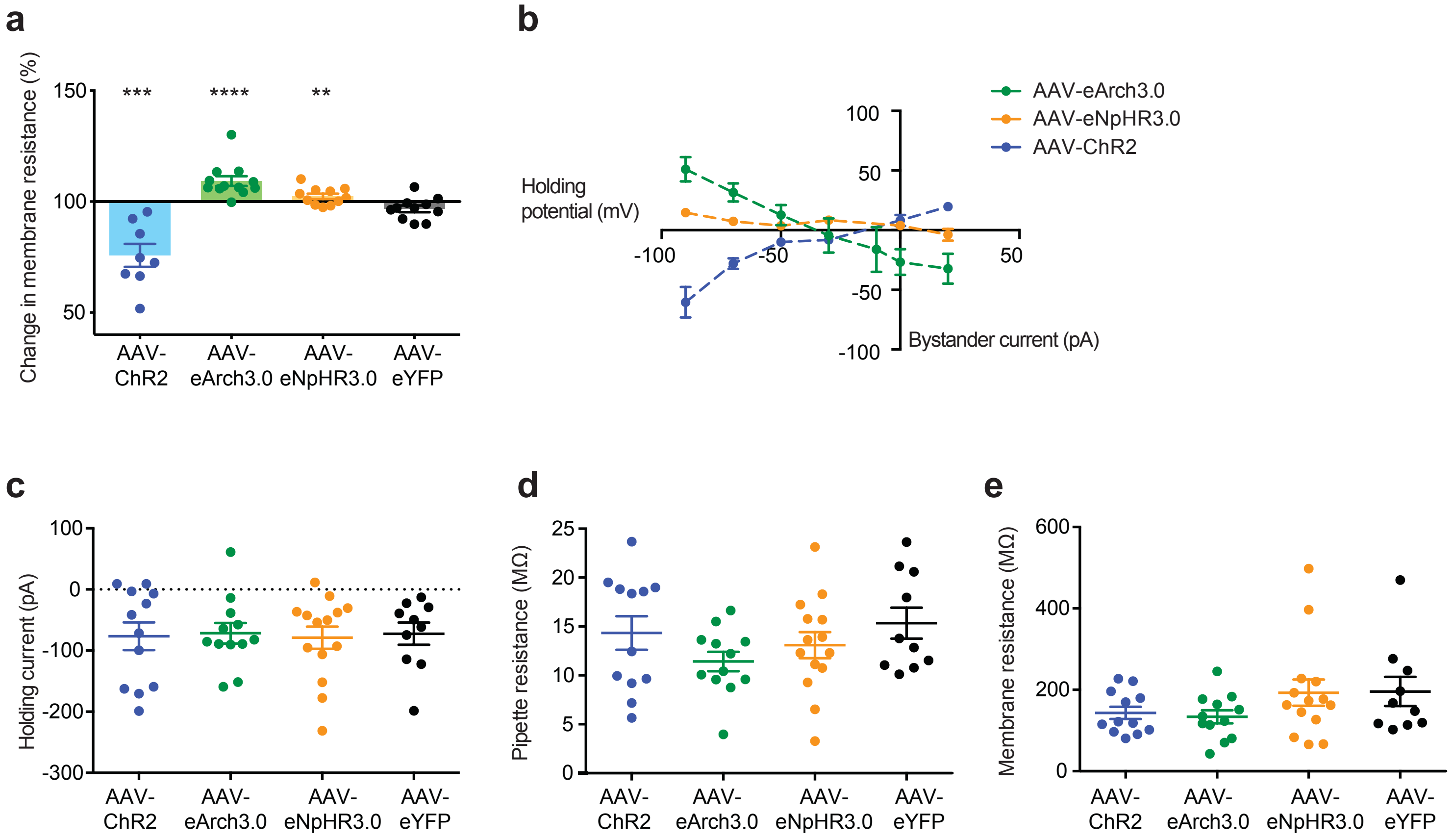

Supplementary Figure 10

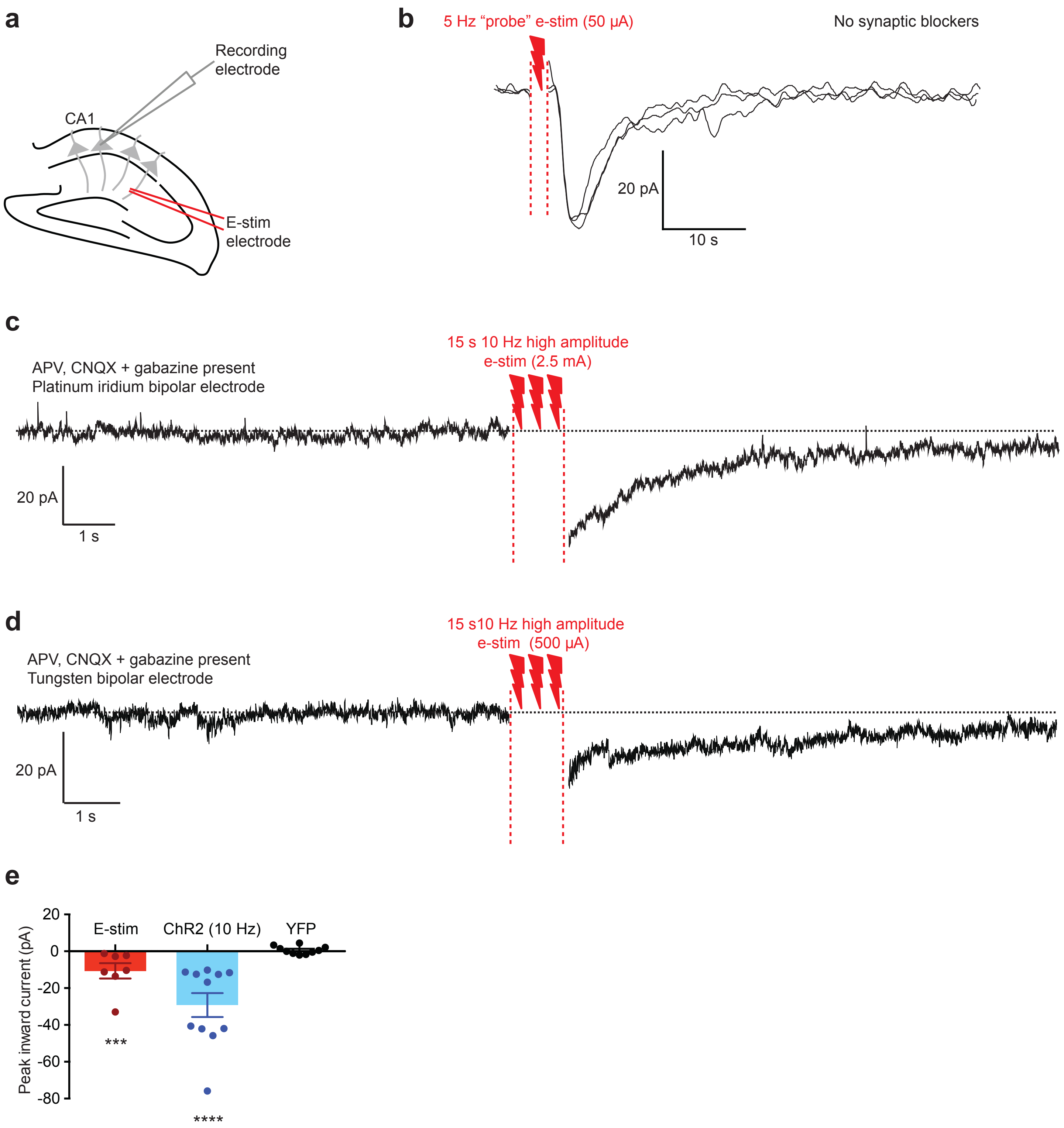

Supplementary Figure 11

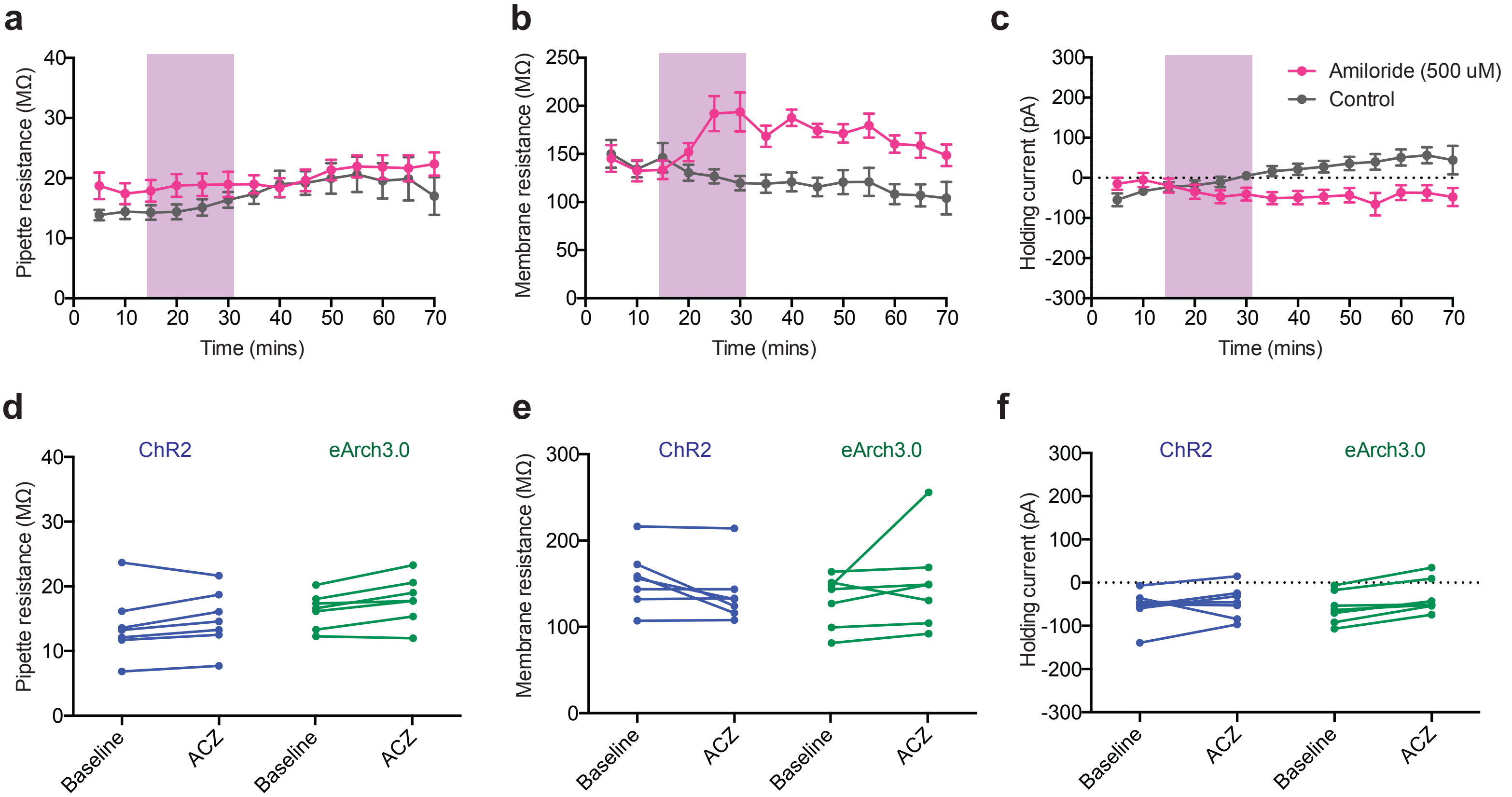

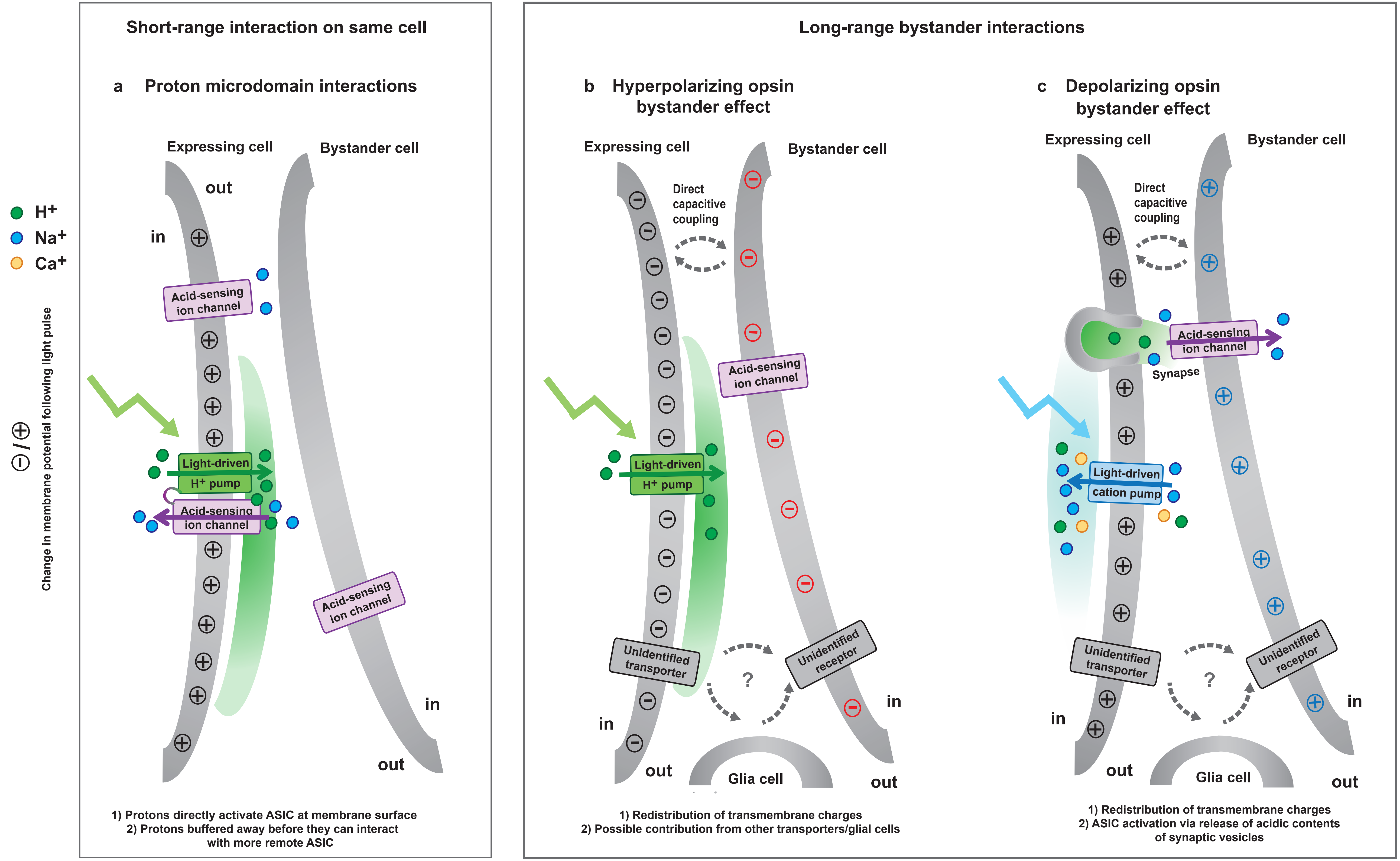
Short-range interaction on same cell
a Proton microdomain interactions
Long-range bystander interactions
b Hyperpolarizing opsin bystander effect
c Depolarizing opsin bystander effect
H+
Na+
Ca+
Change in membrane potential following light pulse
Expressing cell
Bystander cell
out
in
Acid-sensing ion channel
Light-driven H+ pump
Light-driven cation pump
Direct capacitive coupling
Synapse
Unidentified transporter
Unidentified receptor
?
Glia cell
1) Protons directly activate ASIC at membrane surface
2) Protons buffered away before they can interact with more remote ASIC
1) Redistribution of transmembrane charges
2) Possible contribution from other transporters/glial cells
1) Redistribution of transmembrane charges
2) ASIC activation via release of acidic contents of synaptic vesicles
